# Atomic-scale origin of the low grain-boundary resistance in perovskite solid electrolyte Li_0.375_Sr_0.4375_Ta_0.75_Zr_0.25_O_3_

**DOI:** 10.1038/s41467-023-37115-6

**Published:** 2023-04-06

**Authors:** Tom Lee, Ji Qi, Chaitanya A. Gadre, Huaixun Huyan, Shu-Ting Ko, Yunxing Zuo, Chaojie Du, Jie Li, Toshihiro Aoki, Ruqian Wu, Jian Luo, Shyue Ping Ong, Xiaoqing Pan

**Affiliations:** 1 Department of Materials Science and Engineering, University of California at Irvine, Irvine, CA USA; 2 Materials Science and Engineering Program, University of California San Diego, La Jolla, CA USA; 3 Department of Physics and Astronomy, University of California at Irvine, Irvine, CA USA; 4 Department of NanoEngineering, University of California San Diego, La Jolla, CA USA; 5 Irvine Materials Research Institute, University of California at Irvine, Irvine, CA USA

**Keywords:** Batteries, Imaging techniques, Computational science, Batteries

## Abstract

Oxide solid electrolytes (OSEs) have the potential to achieve improved safety and energy density for lithium-ion batteries, but their high grain-boundary (GB) resistance generally is a bottleneck. In the well-studied perovskite oxide solid electrolyte, Li_3*x*_La_2/3-*x*_TiO_3_ (LLTO), the ionic conductivity of grain boundaries is about three orders of magnitude lower than that of the bulk. In contrast, the related Li_0.375_Sr_0.4375_Ta_0.75_Zr_0.25_O_3_ (LSTZ0.75) perovskite exhibits low grain boundary resistance for reasons yet unknown. Here, we use aberration-corrected scanning transmission electron microscopy and spectroscopy, along with an active learning moment tensor potential, to reveal the atomic scale structure and composition of LSTZ0.75 grain boundaries. Vibrational electron energy loss spectroscopy is applied for the first time to reveal atomically resolved vibrations at grain boundaries of LSTZ0.75 and to characterize the otherwise unmeasurable Li distribution therein. We find that Li depletion, which is a major reason for the low grain boundary ionic conductivity of LLTO, is absent for the grain boundaries of LSTZ0.75. Instead, the low grain boundary resistivity of LSTZ0.75 is attributed to the formation of a nanoscale defective cubic perovskite interfacial structure that contained abundant vacancies. Our study provides new insights into the atomic scale mechanisms of low grain boundary resistivity.

## Abstract (web summary)

Oxide solid electrolytes generally suffer from high grain boundary resistance. Here, the authors use advanced electron microscopy, along with an active learning moment tensor potential, to reveal the atomic-scale origin of low grain-boundary resistance in Li_0.375_Sr_0.4375_Ta_0.75_Zr_0.25_O_3_.

## Introduction

As the world transitions toward powering itself solely on sustainable sources of energy, energy storage devices become a critical component in making this process a reality. Among these devices, secondary lithium-ion batteries (LIBs) have been regarded as the most dominant and promising energy storage device because of their high energy density, high power density, long cycle life, and low self-discharge. The standard liquid carbonate-based electrolytes of current commercial LIBs still suffers from a few drawbacks, including the safety concerns of their thermal stability and leakage^1^. Solid-state electrolytes, on the other hand, are intrinsically safer and leak free. As an important component of all-solid-state lithium-ion batteries (ASSBs), solid-state electrolytes can enable the use of lithium metal anode and dramatically increase the energy density of LIBs^2^. Nevertheless, numerous challenges, such as poor interfacial contact with electrodes for inorganic solid electrolyte^3^ and unsatisfactory thermal stability for polymer solid electrolyte^4,5^, still stand in the way of employing solid electrolytes to realize competitive ASSBs.

Among the various solid electrolytes, oxide-based ones have drawn significant interest because of their electrochemical, thermal, and structural stability. One bottleneck that limits most oxide solid electrolytes from achieving high total ionic conductivity is the large grain-boundary (GB) resistance^6–14^. Many lithium-ion conductors have bulk ionic conductivity that are high enough for building practical ASSBs^7^. However, their total ionic conductivity is typically orders of magnitude lower than the bulk ionic conductivity due to large GB resistance^7^. One of the most well-studied materials that exhibits this issue is the perovskite-type solid electrolyte Li_3*x*_La_2/3-*x*_TiO_3_ (LLTO, 0 < *x* < 0.16). In its first report by Inaguma and coworkers, LLTO displayed a high bulk ionic conductivity (*σ*_b_) of 1.0 × 10^−3^ S/cm at room temperature. But because its apparent GB ionic conductivity (*σ*_gb_) was 7.5 × 10^−5^ S/cm, the total ionic conductivity (*σ*_t_) was 7 × 10^−5^ S/cm at room temperature^15^. Subsequent study revealed that the GBs comprised of severe structural and chemical deviation of about 2–3 unit cells thick, essentially forming a nanoscale TiO_2_-like insulating interfacial phase^16,17^. Such GBs prohibit the abundance and transport of charge carrier Li^+^. Nonetheless, few materials are exceptions where the bulk resistance, rather than GB resistance, is the bottleneck that limits the total ionic conductivity. They include perovskite Li_0.375_Sr_0.4375_Ta_0.75_Zr_0.25_O_3_^18–21^, Li_0.375_Sr_0.4375_Ta_0.75_Hf_0.25_O_3_^22^, Li_0.38_Sr_0.44_Ta_0.7_Hf_0.3_O_2.95_F_0.05_^23^, and Li_0.073_La_0.31_NbO_3_^24^ as well as garnet Li_5+*x*_Ba_*x*_La_3-*x*_Ta_2_O_12_^25^ and Li_7_La_3_Zr_2_O_12_^26,27^.

Study of the GB microstructures of these atypical oxide solid electrolytes is important, as it will unravel the origin of their low GB resistance and provide insights to overcome the ubiquitous bottleneck of high GB resistance in other materials. To allow for a straightforward comparison with literature results on LLTO, we select perovskite oxide solid electrolyte Li_0.375_Sr_0.4375_□_0.1875_Ta_0.75_Zr_0.25_O_3_ (denoted as LSTZ0.75, where 0.75 refers to the value of *y* in the general formula Li_0.5*y*_Sr_1-0.75*y*_□_0.25*y*_Ta_*y*_Zr_1-*y*_O_3_ and the □ represents the A-site vacancy) for study. This material achieved a *σ*_b_ of 3.5 × 10^−4^ S/cm, a rather high apparent *σ*_gb_ of 1.2 × 10^−3^ S/cm (including the grain size effect), and a *σ*_t_ of 2.7 × 10^−4^ S/cm at room temperature^18^. It is unknown why LSTZ and LLTO show different trends in grain boundary conduction even though they have the same perovskite (ABO_3_) crystal structure. Neither the specific GB ionic conductivity ${\sigma }_{{{{{{\rm{gb}}}}}}}^{{{{{{\rm{spec}}}}}}}$, which represents the intrinsic conduction behavior of the GB, nor the atomic scale microstructures of LSTZ GBs and grain bulk have been measured or characterized previously. Nevertheless, it is reasonable to expect the specific GB ionic conductivity of LSTZ is higher than those of LLTO and other Li-ion conductors because of the rather high *σ*_gb_. As for the LSTZ grain bulk, its microstructural study will shed light on how to increase the bulk, and therefore the total, ionic conductivity. Finally, the atomic scale characterization of Li^+^ ion distribution is important for solid electrolyte materials. Previous studies have shown distinct Li-K edges were difficult to identify in perovskite-type solid electrolytes, owing to their lower volume densities of Li^+^ ions compared to other types of electrolytes^28,29^. Thus, a technique other than conventional low energy core-loss electron energy loss spectroscopy (EELS) is needed.

Computational studies of complex GB structures have also been constrained by the tradeoff between accurate, but expensive ab initio methods^30–35^ and cheap, but inaccurate classical interatomic potentials (IAPs)^36^. In recent years, IAPs that utilize machine learning (ML) to map the potential energy surface to local environment descriptors have emerged as a promising alternative that combines high accuracy and speed^36–42^. Nevertheless, studies of GB structures with ML-IAPs remain challenging due to the more complex local environment at GB regions^39,43^, with most studies focusing on low-sigma GB models^44,45^, even though high-sigma and general GBs are frequently observed in experiments.

Herein, we present an atomic scale study of LSTZ0.75 to reveal the origin of its low GB resistivity. Aberration-corrected scanning transmission electron microscopy (STEM) in combination with EELS show that GBs in LSTZ0.75 consist of defective perovskite structures with significant amounts of vacancies. Furthermore, state-of-the-art vibrational EELS technique is employed for the first time to map atomic-scale vibrations at grain boundaries and determine the otherwise unmeasurable Li distribution in GBs of LSTZ0.75. Its result shows that Li^+^ concentration at GBs is the same as that in grain bulk, indicating GBs do not suffer from the detrimental Li^+^ depletion. Using an efficient active learning strategy, a moment tensor potential (MTP) for LSTZ0.75 was developed to accurately model both bulk and GB structures. Hybrid Monte Carlo/molecular dynamics (MC/MD) simulations corroborate the existence of Sr vacancies in both low- and high-sigma GBs, leading to comparable Li^+^ diffusivity at the GB and bulk regions. Additionally, these simulations indicate that disruption of A-site ordering in the grain bulk of LSTZ0.75 will result in higher bulk ionic conductivity. Our results provide a new insight of the atomic scale origin of the low GB resistivity in LSTZ0.75, which can help us to design and optimize other solid electrolytes. In a broader perspective, the vibrational EELS approach presented here should be applicable to perovskite oxide solid electrolytes in general as well as any LIB material in which distinct Li-K edges cannot be convincingly identified via conventional low energy core-loss EELS. Similarly, our presented active learning workflow we applied here should be generalizable to other complex structures beyond the scale of Ab initio molecular dynamics (AIMD) simulations.

## Results

### Structural ordering in LSTZ grain bulk and its effect on bulk ionic conductivity

**Fig. 1:**
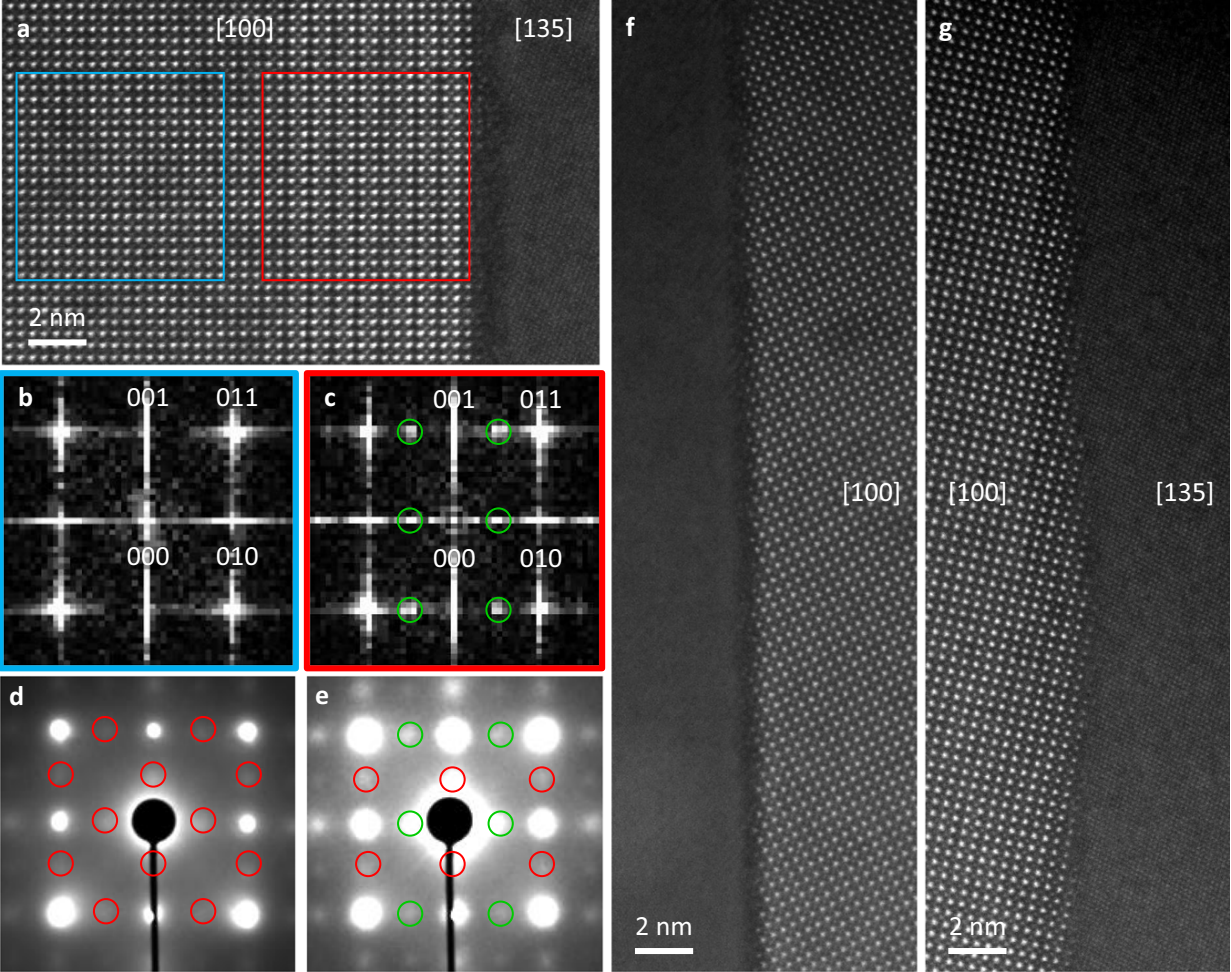
Atomic-scale study of the crystal structure inside the grain bulk, a (010) faceted grain boundary (GB), and general GBs. **a** Atomic-resolution HAADF-STEM image of a (010) faceted GB (with respect to the left-side grain). The zone axes parallel with the incident electron beam were indicated in (**a**), (**f**), and (**g**). **b** FFT pattern of the crystal structure inside the grain, boxed in blue in (**a**), and **c**, FFT pattern of superlattice structure near (010) faceted GB, boxed in red in (**a**). The $(0\pm \frac{1}{2}1)$, $(0\pm \frac{1}{2}0)$, and $(0\pm \frac{1}{2}-1)$ superlattice spots associated with the long coherence ordering in the (010) plane were circled in green. **d** SAED pattern of LSTZ0.75 grain bulk along the [100] zone axis. The $(0\pm \frac{1}{2}1)$, $(0\pm \frac{1}{2}0)$, $(0\pm \frac{1}{2}-1)$, $(0-1\pm \frac{1}{2})$, $(0\,0\pm \frac{1}{2})$, and $(01\pm \frac{1}{2})$ diffraction spots failed to be observed in the corresponding FFT pattern were circled in red. **e** SAED pattern of superlattice structure near (010) faceted GB along the [100] zone axis. The $(0-1\pm \frac{1}{2})$, $(00\pm \frac{1}{2})$, and $(01\pm \frac{1}{2})$ diffraction spots failed to be observed in the corresponding FFT pattern were circled in red. **f**, **g** Atomic-resolution HAADF-STEM images of general GBs. The left-side grain in (**f**) was not oriented along any particular zone axis.

The LSTZ0.75 ceramics were synthesized via conventional solid state reaction method. Results from crystal structure, ionic conductivity, and electrochemical stability characterization (Supplementary Fig. 1) demonstrate that our as-synthesized LSTZ0.75 ceramics have bulk properties similar to those reported in literature. A detailed analysis of these results is in the supplementary information under the section titled crystal structure, ionic conductivity, and electrochemical stability. Figure 1a displays the atomic-resolution high-angle annular dark-field (HAADF) STEM image of a (010) faceted GB in LSTZ0.75. For the left-side grain in [100] zone axis, there is an alternate stacking between bright and dark A-site atomic columns near the GB that persists approximately 17 unit cells into the grain interior. Figure 1b, c displays the fast Fourier transform (FFT) pattern of the region boxed in blue and red, respectively. Only primary spots corresponding to the disordered cubic perovskite structure are observed in Fig. 1b. However, besides the primary spots, one set of superlattice spots arose from the A-site alternate stacking is also prominent in Fig. 1c. This set of superlattice spots is associated with the ordering in the (010) plane. The representative $(0\pm \frac{1}{2}1)$, $(0\pm \frac{1}{2}0)$, and $(0\pm \frac{1}{2}-1)$ spots were circled in green. From the analysis of FFT patterns, it appears that only ordering with long coherence length is present in the region near GB. However, FFT patterns lose phase information and may overlook certain features. Low magnification HAADF-STEM image (Supplementary Fig. 2) shows numerous dark spots in the grain interior of both grains which can be either a local clustering of point defects or structural ordering with extremely short coherence length. The former would not exhibit superlattice spots in electron diffraction, while the latter would. In order to thoroughly understand this feature, selected area electron diffraction (SAED) in TEM is conducted. Figure 1d shows SAED pattern of LSTZ0.75 grain bulk along the [100] zone axis. Surprisingly, the SAED collected from grain bulk exhibits two sets of diffraction spots that had failed to be observed in the corresponding FFT pattern. Twelve representative spots from these two sets of diffraction spots were circled in red. Figure 1e shows SAED pattern of superlattice structure near (010) faceted GB along the [100] zone axis. The SAED from A-site alternate stacking similarly exhibits an additional set of diffraction spots that was not revealed in the corresponding FFT pattern. Six representative spots from the additional set of diffraction spots were circled in red.

Results from SAED reveal the existence of short coherence length ordering in LSTZ0.75 grain bulk. As for the bulk region near (010) faceted GB, both short and long coherence length ordering were present, though the short coherence length ordering in the (001) plane is masked by the long coherence length ordering in the (010) plane. The short coherence length ordering observed here is similar to the mesoscopic coherence length ordering observed in cubic LLTO^46^. Both features could not be detected by XRD due to the short length scale. However, the mesoscopic ordering in cubic LLTO can be detected by FFT pattern (and no SAED was performed to confirm), whereas the short coherence length ordering in cubic LSTZ was overlooked by FFT pattern and can only be detected using SAED.

**Fig. 2:**
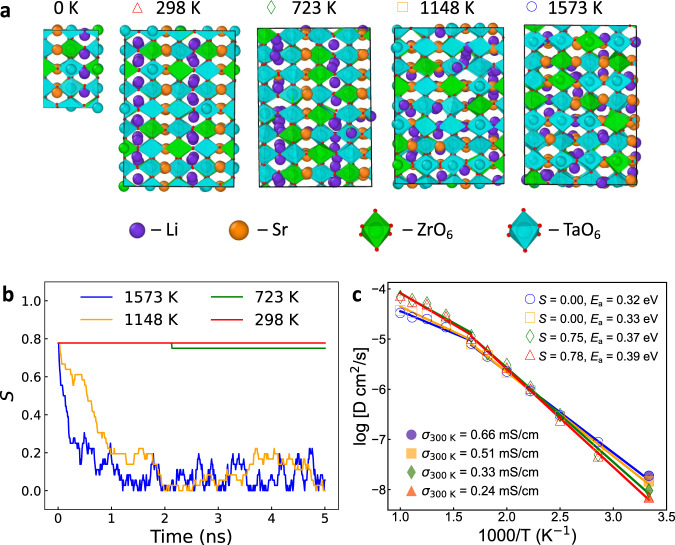
The temperature-dependent A-site ordering in bulk LSTZ0.75 and its effect on Li^+^ diffusion. **a** Crystal structure of LSTZ0.75 unit cell with the most stable ordering by DFT at 0 K and the 2 × 2 × 2 supercells equilibrated by MC/MD with MTP at 298 to 1573 K with 425 K intervals. **b** The evolution of A-site ordering parameter *S* during the 5 ns MC/MD simulations at the four temperatures. The A-site ordering parameter is given by $S=\frac{{R}_{{{{{{\rm{Sr}}}}}}-{{{{{\rm{rich}}}}}}}-{R}_{{{{{{\rm{Sr}}}}}}-{{{{{\rm{overall}}}}}}}}{1-{R}_{{{{{{\rm{Sr}}}}}}-{{{{{\rm{overall}}}}}}}}$, where ${R}_{{{{{{\rm{Sr}}}}}}-{{{{{\rm{rich}}}}}}}$ and ${R}_{{{{{{\rm{Sr}}}}}}-{{{{{\rm{overall}}}}}}}$ refer to A-site occupancy by Sr in Sr-rich layers and the overall A-site occupancy by Sr in LSTZ. For LSTZ0.75, *S* ranges from 0 (complete disorder) to 0.78 (all Sr in Sr-rich layers). **c** The Arrhenius plot of the four models equilibrated at different temperatures. The room temperature ionic conductivity (*σ*_300K_), *S* and activation energy (*E*_a_) below 600 K were listed.

The structural ordering and local composition were further elucidated using a moment tensor potential (MTP) that has been fitted to accurately reproduce the DFT potential energy surface of both bulk and GB structures of LSTZ0.75 using an active learning scheme (see Supplementary Fig. 3 and “Methods”). Hybrid Monte Carlo/molecular dynamics (MC/MD) simulations were performed on a 2 × 2 × 2 supercell at four different temperatures (298, 723, 1148, and 1573 K). Figure 2a shows evolution of the LSTZ0.75 unit cell as temperature increases. Between 0 and 723 K, LSTZ0.75 is characterized by A-site alternate stacking of Sr-rich and Sr-poor layers (Fig. 2a). The A-site ordering parameter *S* is near its maximum value of 0.78 (Fig. 2b), i.e., nearly all Sr are in the Sr-rich layers. However, this A-site stacking becomes disordered at above 1148 K, and *S* decreases to below 0.2.

MD simulations were performed to the equilibrated structures (Fig. 2a) from the 5 ns MC/MD simulations at the four different temperatures. As shown in the Arrhenius plot in Fig. 2c, simulated bulk ionic conductivities at 300 K (*σ*_300K_) of the two structures equilibrated at 298 K and 723 K with *S* ≈ 0.78 match well with experimentally measured *σ*_b_ at room temperature, while the *σ*_300K_ of the other two structures equilibrated at 1148 K and 1573 K with disordered A-sites (*S* ≈ 0) are promoted by 1–2 times. It was also found that a decrease in *S* leads to lower activation energy (*E*_a_). The increased *σ*_300K_ and the decreased *E*_a_ at *S* ≈ 0 match more closely with the highest observed experimental *σ*_b_ of 4.1 × 10^−4^ S/cm at 25 °C and *E*_a_ of 0.33 eV (calculated from 25 to 140 °C) reported for a hot-pressed LSTZ0.75 sample, whose A-site ordering was not yet characterized^20^. The high *σ*_b_ and low *E*_a_ of this hot-pressed LSTZ0.75 might be attributed to the elimination of A-site alternate stacking, for which future in-depth experimental characterization is deserved. Corroborating with our result, similar trends have been observed in LLTOs, in which higher calcination temperatures lead to a tetragonal to cubic transformation and an increase in *σ*_b_ from ~7 × 10^−4^ S/cm to ~1.5 × 10^−3^ S/cm^47,48^. With the SAED results indicating the existence of short coherence length A-site ordering in LSTZ0.75 grain bulk and the trend uncovered in Fig. 2c, we believe the *σ*_b_ of LSTZ0.75 can be further enhanced by promoting disorder in A-sites.

### Microstructure of LSTZ0.75 GBs revealed by STEM-EELS

Results from AC impedance spectroscopy (Supplementary Fig. 1c) shows that ${\sigma }_{{{{{{\rm{gb}}}}}}}^{{{{{{\rm{spec}}}}}}}$ = 7.96 × 10^−7^ S/cm for LSTZ0.75, which is significantly higher than (i.e., ~26× of) ${\sigma }_{{{{{{\rm{gb}}}}}}}^{{{{{{\rm{spec}}}}}}}$ = 3.1 × 10^−8^ S/cm of LLTO^6^. This indicates Li-ion transport in the GBs of LSTZ is indeed significantly faster than that in GBs of LLTO. To reveal origin of the low grain-boundary resistance in LSTZ0.75, detailed characterization of the GBs is performed. Electron Backscatter Diffraction (EBSD) results (Supplementary Fig. 4) indicated that the average grain size is 3.38 ± 1.13 μm, and the GBs mainly consist of randomly orientated grains. In total, we have imaged more than seven hundred GBs, of which ~12% have (010) faceted grain terminal surfaces on one side. As shown in Fig. 1a, an obvious dark band can be observed at the (010) faceted GB. Figure 1f, g exhibits atomic-resolution HAADF-STEM images of general GBs with complex, high-index surfaces. Obvious dark bands at the GBs are still present, while no obvious ordering is observed in the region near GBs. In fact, of the >700 GBs imaged, we have only observed the alternate stacking between bright and dark A-site atomic columns near (010) faceted GBs (~12% of all GBs). However, all of the GBs exhibited a drastic decrease in image intensity in comparison with grain bulk, regardless of the relative orientation between the two adjacent grains. Since the HAADF-STEM image intensity is dictated by the average atomic number of the elements present^49^, this observation clearly indicated a compositional variation across the GB. Specifically, the decrease in intensity at the A-site atomic columns can be attributed to a decrease in concentration of Sr (either an increase in the amount of Li substituting Sr or simply increase in vacancies). Similarly, the decrease in intensity at the B-site atomic columns can be attributed a decrease in concentration of Ta and/or Zr. It is worth noting that despite the compositional change, the cubic perovskite crystal structure is still maintained at the GBs. Supplementary Fig. 5a and b shows high magnification atomic-resolution HAADF-STEM images of the general GB from Fig. 1g and a (010) faceted GB (with respect to the right-side grain), respectively. Since the same crystal lattices from the bulk extend all the way to the GBs, and the only change is the observed decrease in intensity at GBs, crystal structure of the GBs is also cubic perovskite.

**Fig. 3:**
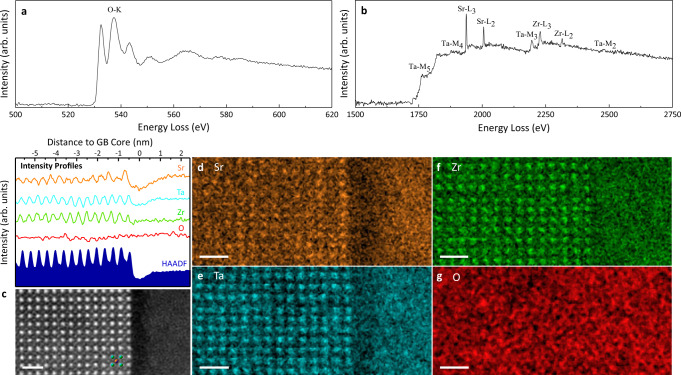
Core-loss EELS data of (010) faceted GB. Integrated EEL spectra of **a** O-K and **b** Sr-L_2,3_, Zr-L_2,3_, Ta-M_2,3_, and Ta-M_4,5_ edges for (010) faceted grain boundary shown in (**c**). **c** Atomic-resolution HAADF-STEM image of a (010) faceted grain boundary (with respect to the left-side grain). Atoms of a single unit cell (A-site centered view) are labeled, in accordance with the schematic of LSTZ crystal structure (Fig. 1b). Elemental maps of **d** Sr, **e** Ta, **f** Zr, and **g** O. All scale bars are 1 nm. Intensity profiles of **c**–**g** are shown above (**c**).

To better understand the observed features in HAADF images, high spatial resolution STEM-EELS measurements were performed at (010) faceted GBs and general GBs. Figure 3a, b shows the integrated EEL spectra of O-K edge and edges in the high energy loss regime (Sr-L_2,3_, Zr-L_2,3_, Ta-M_2,3_, and Ta-M_4,5_ edges), respectively. Atomic-resolution HAADF-STEM image of the (010) faceted GB from which the EEL spectra were collected is shown in Fig. 3c. The GB again exhibited a dramatically lower image intensity in comparison with the bulk. For the left-side grain, the alternate stacking between bright and dark A-site atomic columns, which extends ten unit cells from GB into grain interior, is also observed. Figure 3d–g displays the atomic-resolution elemental maps of Sr, Ta, Zr, and O, respectively. The maps were generated using intensities from Sr-L_2,3_, Zr-L_2,3_, Ta-M_4,5_, and O-K edges. As expected, the elemental maps (Fig. 3d–f) show Sr atoms reside in the A-site positions, and Ta/Zr atoms reside in the B-site positions. Atoms of a single unit cell are labeled, in accordance with the schematic of LSTZ0.75 crystal structure (Supplementary Fig. 1a), in Fig. 3c. Bright field (BF) STEM image (Supplementary Fig. 6b) further confirms that, in addition to overlapping with B-site atoms, O atoms also reside between two adjacent B-site atoms. In summary, the STEM images and EELS elemental maps confirmed the proposed schematic of LSTZ0.75 crystal structure (Supplementary Fig. 1a) to be correct.

To analyze the correlation between HAADF image and the elemental maps, a plot of vertically integrated intensity profiles across the GB is shown above the HAADF image (Fig. 3c). For the GB, the dramatic decrease in image intensity correlates well with the decrease in intensity of Sr, Ta, and Zr signals, though the decrease in Zr signal is much less than the other two. Specifically, there is a ~26%, 11%, and 7% decrease in Sr, Ta, and Zr signals, respectively, at the GB when compared to those from the abutting grains. This indicates that the GB contains more Sr, Ta, and Zr vacancies than the grain bulk, and its crystal structure resembles that of a defective cubic perovskite. Further analysis reveals that higher Sr and Ta signals are observed at the left side adjacent to the GB. For the left-side grain, variation in HAADF intensity correlates reasonably well with that in Sr elemental map intensity. The bright A-site atomic columns in superlattice (SL) region contains more Sr than those in other regions of the grain and can be considered as Sr-rich layer. By the same token, the first dark A-site atomic column immediately adjacent to the GB core contains less Sr than A-site atomic columns in other regions of the grain and can be considered as a single Sr-poor layer. The remaining dark A-site atomic columns contain approximately the same amount of Sr as those in other regions of the grain. Analysis of the Sr signals indicates that this SL region contains more Sr than the rest of grain bulk. In other words, this region has a higher density, and the A-site vacancies are filled with Sr. This decrease of A-site vacancies will decrease the number of Li percolation pathways and impede Li^+^ ion migration. Therefore, from both structural ordering and concentration of A-site vacancy point of view, the superlattice structure is unfavorable for achieving high bulk ionic conductivity. Nonetheless, since the (010) faceted GBs only constitute ~12% of all GBs, the impact such structure has on the overall bulk ionic conductivity should be insignificant. Finally, slightly higher Ta signals can be observed in the three B-site atomic columns located at −0.5, −0.9, and −1.3 nm distance to GB. The results above indicate that the GB has undergone elemental segregation, with Sr and Ta segregates out of the GB core and into side of the left grain. The increased amount of cation vacancies at GB provides more Li percolation pathways, which will facilitate Li^+^ ion migration and decrease GB resistance. Unlike the intensity of Sr, Ta, and Zr signals, no decrease is observed in the intensity of O signals at the GB. Finally, STEM imaging coupled with EELS measurements were also performed at general GBs and results are shown in Supplementary Figs. 7, 8. A similar trend is observed in the atomic-resolution HAADF image and elemental maps of Sr, Ta, Zr, and O. Both general GBs contain Sr, Ta, and Zr vacancies. When compared with the (010) faceted GB, Ta vacancies, instead of Zr vacancies, are much less than Sr and Zr vacancies at these 2 general GBs. For the GB shown in Supplementary Fig. 7, elemental segregation of Sr and Ta can be observed at both sides adjacent to the GB, while that of Zr is observed mostly in the grain to the left of GB. As for the GB shown in Supplementary Fig. 8, elemental segregation of Sr and Zr can is seen at both sides adjacent to the GB, while that of Ta is observed mostly in the left grain. The differences among the (010) faceted GB and 2 general GBs can be attributed to subtle variation in the composition of GBs. Consistent with the lack of ordering observed in the HAADF-STEM images (Fig. 1f, g), the Sr elemental maps did not exhibit alternate stacking of A-site.

### LSTZ0.75 GB composition and its effect on ionic conductivity revealed by MTP

**Fig. 4:**
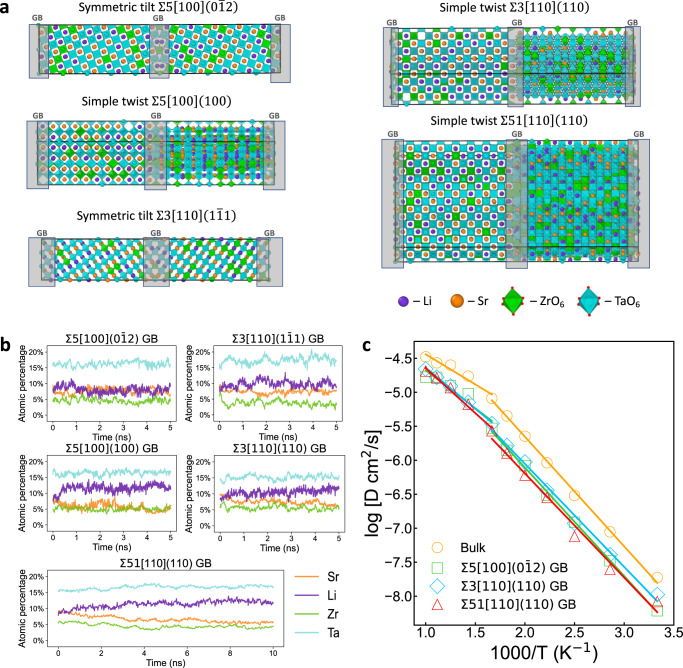
Local composition and Li^+^ diffusion at low-sigma and high-sigma GBs. **a** The structures of the four low-sigma and the one high-sigma GB models, where the GB orientations and the GB regions located at the center and edge of the cells were labeled. GB thickness was defined as two or four times of the interplanar distance between GB planes with a general requirement of *d*_GB_ > 5 Å. The exact geometric information of those GB models was provided in Supplementary Table 3. **b** The evolution of the atomic percentage of cations at GB regions of the five GB models during the MC/MD at 1573 K. **c** The Arrhenius plot of Li^+^ diffusivity calculated using the bulk model, the two most stable low-sigma GBs and the high-sigma GB. The GB models were equilibrated with MC/MD simulations at 1573 K. The respective *D*_Li, 300 K_ and *E*_a_ were provided in Table 2. The Arrhenius plot of GB models without equilibration was provided as Supplementary Fig. 9.

The relationship between GB composition and ionic conductivity was probed using the MTP. Specifically, four low-sigma GBs (symmetric tilt $\Sigma 5\left[100\right](0\bar{1}2)$, simple twist $\Sigma 5\left[100\right]\left(100\right)$, symmetric tilt $\Sigma 3\left[110\right]\left(1\bar{1}1\right)$, and simple twist $\Sigma 3\left[110\right]\left(110\right)$) and one high-sigma (simple twist $\Sigma 51[110](110)$) GBs were used as model systems (Fig. 4a). It is worthy to mention that sigma 3 and 5 GBs have been identified to be the two most frequent sigma values for low-sigma GBs by EBSD analysis (Supplementary Fig. 4e), and the simulated GB angles (Supplementary Table 3) also covered the measured distribution of misorientation angles (Supplementary Fig. 4d). Meanwhile, our active learning MTP successfully reproduced the DFT GB energies of the low-sigma GBs inside and outside the training set to within 0.10 J/m^2^ (Supplementary Table 4). We thereby are confident to use the MTP to simulate the large-sigma GB that can represent general GB^46^. MC/MD simulations were performed at the experimental calcination temperature of 1573 K. It is found that the atomic percentage of Li^+^ at GB regions increased from around 8% to over 11% in the two simple twist GBs and the high-sigma GB, i.e., $\Sigma 5[100](100)$, $\Sigma 3[110](110)$, and $\Sigma 51[110](110)$ (Table 1, Fig. 4b). This is accompanied by a decrease in the atomic percentage of Sr, with the formation of more Sr vacancies, at the GB regions. From these results, Li^+^ depletion, one of the major causes of low GB conductivity in LLTO^16^, is generally unfavorable in LSTZ, while formation of Sr vacancies at GB regions is favorable, in line with the experimental observations of Sr vacancies at GB regions. To understand the formation of Sr vacancies on LSTZ0.75 GB, the anti-site energies in the $\Sigma 3[110](110)$ GB model is simulated using DFT. As in Supplementary Table 5, the anti-site energy of swapping Li at GB and Sr at bulk is as high as 5.0 eV, while that of swapping Li at bulk and Sr at GB is as low as 3.2 eV, indicating that Li tends to swap to GB region while Sr prefer swapping to bulk region. This thermodynamic tendency explains why there are more Sr vacancies in LSTZ0.75 GB.

**Table 1:** Local composition of the GB regions in the five GB models before and after MC/MD simulations at 1573 K

Element	Sr	Li	Zr	Ta	O
LSTZ0.75	9.1%	7.8%	5.2%	15.6%	62.3%
$\Sigma 5[100](0\bar{1}2)$ GB, ${\gamma }_{{{{{{\rm{GB}}}}}}}$ = 0.67 J/m^2^					
MC/MD 0 ns	9.5%	8.3%	4.8%	15.6%	61.9%
MC/MD 5 ns	6.5%	9.1%	4.5%	16.2%	63.6%
$\Sigma 5[100](100)$ GB, ${\gamma }_{{{{{{\rm{GB}}}}}}}$ = 1.01 J/m^2^					
MC/MD 0 ns	8.4%	7.6%	5.0%	16.0%	63.0%
MC/MD 5 ns	6.1%	11.3%	5.2%	16.9%	60.5%
$\Sigma 3[110](1\bar{1}1)$ GB, ${\gamma }_{{{{{{\rm{GB}}}}}}}$ = 0.98 J/m^2^					
MC/MD 0 ns	9.2%	8.9%	6.0%	14.7%	61.2%
MC/MD 5 ns	7.9%	8.7%	3.2%	17.2%	63.0%
$\Sigma 3[110](110)$ GB, ${\gamma }_{{{{{{\rm{GB}}}}}}}$ = 0.72 J/m^2^					
MC/MD 0 ns	9.7%	8.3%	5.7%	14.8%	61.5%
MC/MD 5 ns	5.7%	11.7%	5.1%	15.7%	61.8%
$\Sigma 51[110](110)$GB, ${\gamma }_{{{{{{\rm{GB}}}}}}}$ = 3.69 J/m^2^					
MC/MD 0 ns	8.7%	7.9%	5.5%	15.3%	62.6%
MC/MD 10 ns	5.7%	11.7%	4.4%	16.6%	61.9%

The atomic percentage of LSTZ0.75 was listed for reference.

MD simulations were conducted to the two most stable low-sigma GBs (symmetric tilt $\Sigma 5\left[100\right]\left(0\bar{1}2\right)$, simple twist $\Sigma 3[110](110)$) and the high-sigma GB. As shown in Fig. 4c, non-Arrhenius behaviors with transition temperatures of around 600 K can be generally observed, similar to what has been observed for LLTO^15,50^. However, the GB Li^+^ diffusivities *D*_Li, 300 K_ of LSTZ0.75 are only 2–3 times lower than those of bulk LSTZ0.75 (Table 2). This is in sharp contrast to LLTO, where it has been reported that the GB ionic conductivity is 1–2 orders of magnitude lower than bulk ionic conductivity^15,51^. The GB activation energies in LSTZ0.75 are also comparable to the bulk value, while in LLTO, the GB activation energies are generally higher than bulk^51^. Furthermore, we note that the non-equilibrated GBs have a higher *E*_a_ and lower *D*_Li, 300 K_ (Table 2, Supplementary Fig. 9), which indicates that Li enrichment and Sr vacancies play a role in increasing the GB Li^+^ diffusivity. We also analyzed the directional *D*_Li_ perpendicular to (*D*_⊥GB_) and parallel to (*D*_//GB_) the GB planes, which are all comparable to the overall *D*_Li_, indicating fast Li diffusion along and across GB (Table 2). Most importantly, these trends were consistently observed in the low-sigma GBs and the high-sigma GB, indicating fast Li^+^ diffusion at low-sigma GBs as well as general GBs of LSTZ0.75. We note that these general observations remain valid with and without equilibration.

**Table 2:** Activation energies (*E*_a_) and Li diffusivities at 300 K (*D*_Li, 300 K_) of bulk LSTZ0.75 and at the GB regions of the three GB orientations before and after MC/MD simulations at 1573 K

LSTZ0.75 models	*D*_Li, 300 K_ (10^−9^ cm^2^/s)			*E*_a_ 300–600 K (eV)	*E*_a_ 600–1000 K (eV)
	*D*_overall_	*D*_⊥GB_	*D*_//GB_		
Bulk	18.80			0.32	0.18
$\Sigma 5[100](0\bar{1}2)$ GB					
MC/MD 0 ns	5.83	7.60	4.95	0.35	0.21
MC/MD 5 ns	6.11	5.23	6.55	0.32	0.24
$\Sigma 3[110](110)$ GB					
MC/MD 0 ns	6.16	6.37	6.05	0.32	0.22
MC/MD 5 ns	10.58	10.23	11.27	0.30	0.24
$\Sigma 51[110](110)$ GB					
MC/MD 0 ns	7.17	10.06	5.43	0.32	0.26
MC/MD 10 ns	8.34	11.25	6.88	0.30	0.26

### Li distribution in LSTZ0.75 microstructure revealed by vibrational EELS

Although attempts were made to investigate the Li distribution at the GBs by identifying the Li-K edge, the low Li content in LSTZ0.75, poor scattering power of Li (*Z* = 3), and overlap with the Ta-O_2,3_ edge makes this investigation challenging. More detailed results and discussion are reported in Supplementary Fig. 10 and in supplementary information under section titled challenge in determining Li distribution in LSTZ0.75 microstructure by conventional low energy core-loss EELS. Representative spectra in Supplementary Fig. 11a hardly shows distinguishable features for Li-K edge and is overwhelmed by the Ta-O_2,3_ edges due to larger quantities of Ta. Thus, the variation of the average peak height indicated in Supplementary Fig. 11b includes a combination of Ta-O_2,3_ and Li-K edge signals. The line profile and contour plots (Supplementary Fig. 11b, d, respectively) show two small humps in the left grain owing to the SL and a dip at the GB which may suggest Ta or Li vacancies but ambiguous as to which is most responsible for the decrease in signal. As a result, mapping of the Li distribution in grains and GBs of LSTZ0.75 via the Li-K edge is not feasible in systems which contain a large Ta:Li ratio.

To resolve this issue, we map the Li distributions by probing Li-O vibrations. We employ a state-of-the-art vibrational EELS technique enabled by high energy resolution monochromation that has facilitated the spectroscopy of vibrational excitations at the nanometer^53,54^ and even atomic scales^52,55^. We employ a dark field vibrational EELS (DF VibEELS) beam-detector geometry (Supplementary Fig. 12c) to ensure only high spatial resolution signals are acquired^52,56^ and map the atomically-resolved vibrational structure at grain boundaries.

**Fig. 5:**
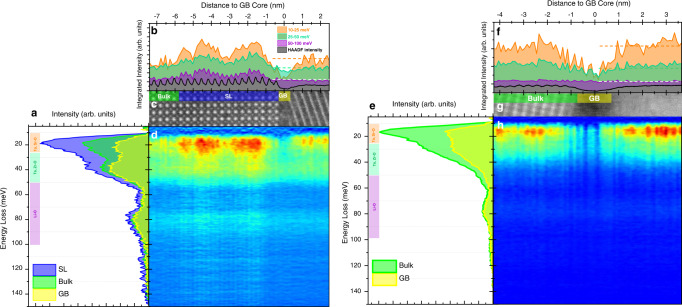
Vibrational EELS of (010) faceted and general GBs. **a** Representative vibrational spectra at the SL, bulk, and GB regions of a (010) faceted GB. Averaged vibrational spectral intensities are higher at the SL region than in the bulk and GB. The colored labeling to the left of the peak denotes the energy ranges over which the signal was averaged and corresponds to specific elemental vibrations with oxygen. **b** Line profiles of the averaged intensities denoted in (**a**) overlaid with a vertically integrated HAADF line profile. While all intensity-averaged curves show modulations in the SL region, only the intensity of the Ta-, Sr-O vibrations shows a large dip at the GB. The B-site vibrational intensity (Ta-, Zr-O) shows a smaller dip while the vibrational EELS signal corresponding to Li-O vibrations shows no change from bulk at the GB. **c** HAADF image corresponding to the region the (010) faceted GB spectra were acquired. **d** Contour plot of vertically-integrated spectral slices stacked horizontally. The vertical axis denotes energy loss while the horizontal axis denotes the horizontal real-space position labeled in the horizontal axis of (**b**). **e** Representative vibrational spectra at the bulk and GB regions of a general GB. **f** Line profiles of the averaged intensities denoted in (**e**) overlaid with a vertically integrated HAADF line profile. The intensity of the Ta-, Sr-O vibrations show a large dip at the GB, the B-site vibrational intensity (Ta-, Zr-O) shows a smaller dip, while the vibrational EELS signal corresponding to Li-O vibrations shows no change from bulk at the GB. Plot labels are the same as in (**b**). **g** HAADF image corresponding to the region the general GB spectra were acquired. **h** Contour plot of vertically-integrated spectral slices stacked horizontally. The streaks in **d**, **h** indicate atomic resolution vibrational mapping^52^.

Representative background-subtracted spectra of LSTZ in the (010) faceted grain interior, grain boundary, and SL region spanning 10–150 meV (Fig. 5a) are consistent with the Raman spectrum of the LSTZ pellet (Supplementary Fig. 13c). Heavier elements in the A- and B-sites, namely Sr, Ta, and Zr, contribute to lower energy vibrations with O and comprise of the low energy peaks from 10 to 50 meV while the lighter Li vibrates with O at a higher frequency and produces vibrations in the 50 to 100 meV range (Supplementary Fig. 13b). Several striking features can be observed in the contour plot (Fig. 5d), including a strong variation in intensity in the SL region and a drastic decrease in intensity at the GB in the low energy region of the phonon signal. The orange curve in Fig. 5b, containing mainly Sr, Ta vibrations with O (Supplementary Fig. 13b), shows strong intensity modulation in the SL region due to the increased concentration of Sr in the SL region while the dip at the GB suggests deficiencies in Sr, Ta vibrational species. Given that the concentration of O is constant throughout this region (Fig. 3g), the DF VibEELS mapping (Fig. 5b) shows Sr and Ta vacancies and is consistent with Fig. 3d–f. The green curve represents the spatial trend of vibrations that are largely dominated by Ta-, Zr-O. Here the dip at the GB is less drastic owing to slight vacancies in Ta and Zr which can be more clearly seen in Fig. 5d. A similar contrast for Ta-, Zr-O vibrations in the SL region is seen in Fig. 5b and, to a much lesser degree, in the intensity profiles in Fig. 3, which suggests that DF VibEELS is extremely sensitive to local elemental modulation and can be extended to map minute variations in composition. This is not unlike VibEELS ability to measure structural changes that manifest as defect modes^57^. The purple curve, containing a majority of Li-O vibrations, also shows strong intensity modulation in the SL region produced by an alternating Sr-rich and Li-rich SL. Unlike the other curves, Li-O shows no decrease in integrated intensity at the GB, indicating that there are no Li vacancies at the GB. This can also be seen in the representative spectra in Fig. 5a where the intensities in the 50–100 meV region hardly change between the bulk and the GB. Given there are Sr, Ta, and Zr vacancies, the relative atomic % of Li is higher at the GB, which is consistent with Fig. 4b and Table 1. Coupled with the phonon DOS, DF VibEELS mapping allows us to not only map the distribution of Li but also Sr, Ta, and Zr at the atomic scale.

DF VibEELS was also conducted on the general GB shown in Supplementary Fig. 7 and similar trends in vibrational intensity can be observed (Fig. 5e–h). Like the (010) faceted GB, the general GB also exhibited Sr, Ta, and Zr vacancies as evidenced by Fig. 5f. Intensity of low energy vibrations in the 10–25 meV and 25–50 meV range corresponding to Sr-, Ta-O and Ta-, Zr-O vibrations decreases at GB and is consistent with the elemental mapping in Supplementary Fig. 7. However, the vibrational intensity in the 50–100 meV corresponding to Li-O vibrations remains constant, suggesting that similar to the (010) faceted GB, general GBs also do not contain Li^+^ vacancies. The slight variation in spectral shapes between (010) faceted GB and general GB spectra are a result of the changes to the in-plane vibrations due to the differing zone axes demonstrating that this technique is highly sensitive to crystal orientation as well.

This technique is indispensable for solid electrolytes with compositions that contain large Ta:Li ratios, as the Ta-O_2,3_ edge completely obscures and overwhelms the Li-K edge and makes it impossible to map Li distributions by conventional core-loss EELS. As a result, DF VibEELS technique emerges as a powerful technique that is solely capable of characterizing LSTZ and LSTZ derivatives (e.g., Li_0.375_Sr_0.4375_Hf_0.25_Ta_0.75_O_3_ and Li_0.38_Sr_0.44_Ta_0.7_Hf_0.3_O_2.95_F_0.05_) at the atomic scale. More generally, we demonstrate vibrational EELS as a highly effective tool for characterizing atomic scale structural and compositional inhomogeneities.

With Li^+^ ion charge carriers preserved, cubic perovskite structure maintained, and vacancies added at the GBs, Li^+^ ion transport at the GBs is enhanced in LSTZ0.75. In comparison, Li^+^ ions were depleted, crystal structure was dramatically altered, and no vacancies were created at the GBs for LLTO^12^. As a result, Li^+^ ion transport at the GBs is severely impeded and LLTO has a large GB resistance.

## Discussion

Based on the results presented above, vacancy engineering of the GBs should be a viable approach to increase the GB ionic conductivity of perovskite oxide solid electrolytes. Previous studies have established that ionic conduction in LLTO occurs via the migration of Li^+^ ions to nearby A-site vacancies^58–60^. Our simulation results indicate the ionic conduction mechanism of LSTZ0.75 is similar to that of LLTO. Since LSTZ0.75 maintained the perovskite framework at GBs, the additional Sr vacancies at its GBs naturally increase the number of percolation pathways and facilitate Li-ion transport. Meanwhile, preservation of the structural framework is important, for it prevents the GBs from forming structures that generate new barriers for Li-ion transport. One method to realize such goals might be incorporation of appropriate dopants into crystal lattice. It has been reported that appropriate crystal doping can improve overall ionic conductivity by introducing additional vacancies while preserving the desired crystal structure^27,61–65^. Beyond this particular LSTZ system, our proposed approach should also benefit other Li-ion conductors, as diffusion to nearby structural defects is a common Li-ion conduction mechanism for solid electrolytes^65,66^.

In conclusion, we report an atomic-scale study of LSTZ to reveal its GB structures to understand their effects on Li-ion conductivity. MTP enabled MC/MD simulations on sophisticated LSTZ0.75 supercells show that disruption of A-site ordering in grain bulk results in increased bulk ionic conductivity. Atomic-resolution STEM and EELS analysis was employed to further study the GB structures. Our results demonstrate that even though the GBs contain substantial amounts of A- and B-site vacancies, the perovskite framework is maintained. DF VibEELS was employed to map atomically resolved vibrations at grain boundaries and the atomic scale Li^+^ distribution in LSTZ0.75 for the first time, which showed that Li^+^ concentration at GBs is the same as that inside the bulk phase. MC/MD also reveals a high relative amount of Sr vacancies and Li^+^ ions at the GBs; these Sr vacancies are found to promote Li-ion diffusivity at GBs. Based on these results, we suggest vacancy and defect engineering can effectively improve GB ionic conductivity of solid Li-ion conductors, given that the material’s original structural framework should be maintained. Our study showcases the importance of understanding bulk structural ordering and GB structures in order to improve the macroscopic properties of polycrystalline materials. In addition, we have demonstrated the necessity of using novel, cutting-edge characterization, and modeling methods to uncover GB structures and kinetics that would otherwise be impossible to probe with conventional tools, which can be generally applied to a broad range of other materials.

## Methods

### Experimental methods

#### LSTZ0.75 ceramics synthesis

LSTZ0.75 were synthesized via conventional solid state reaction method. Stoichiometric amounts of Li_2_CO_3_ (99.99%, Sigma-Aldrich), SrCO_3_ (99.9%, ALB Materials Inc.), ZrO_2_ (99.9%, MSE Supplies), and Ta_2_O_5_ (99.9%, ALB Materials Inc.) were ground and mixed with ethanol for 18 h by high energy vertical planetary ball mill (MSE Supplies) with yttria stabilized zirconia milling media at 900 revolution per minute (RPM), and then calcined at 1100 °C for 12 h in air using an alumina crucible. The calcined powders were ground again, and then pressed into pellets at a pressure of 200 MPa by cold isostatic pressing (CIP). The pellets were then covered with the same mother powder. Quantity ratio (wt%) of the pellet sample to mother powder was 1:2. Finally, LSTZ0.75 pellets covered in mother powder were sintered at 1300 °C for 15 h in air using an alumina crucible.

#### Crystal structure, grain size, ionic conductivity, and electrochemical stability characterization

XRD was conducted using Rigaku Smartlab, with Cu Kα radiation (λ = 1.5406 Å). EBSD was performed using FEI Apreo LoVac SEM with Oxford Instruments symmetry EBSD detector. The maps were scanned at 26 nA and 20 kV, with 0.5 µm step size. The grain size and misorientation distribution statistics were calculated from inverse pole figure maps along the sample normal direction. Ionic conductivity was evaluated at 25 °C and frequency from 100 Hz to 40 MHz with applied voltage amplitude of 0.1 V, using a Hewlett-Packard 4194A Impedance Analyzer. Before the ionic conductivity measurements, Ag paste (Ted Pella, Inc.) was brushed on both planar surfaces of the sintered pellet and subsequently heated at 500 °C for 5 min to coat the lithium-ion blocking Ag electrodes.

Electrochemical properties were measured using Princeton Applied Research Parstat MC potentiostat. An electrochemical test cell of Li‖LSTZ0.75 was tested. Pristine LSTZ0.75 pellets were ground into powder using mortar and pestle. The resulting powder was mixed with carbon black and poly(vinylidene difluoride) (PVDF) in a weight percent ratio of 5:3:2, respectively, in n-methyl-2-pyrrolidone (NMP) solvent to prepare the working electrode. The slurry was coated on 15-µm thick copper foil by the doctor blade technique. The resulting LSTZ0.75 working electrode was 1.2 cm in diameter and had a mass loading of 1.27 mg/cm^2^. Metallic Li foils (99.9% metals basis, Alfa Aesar) were used as counter electrode (thickness = 750 μm, diameter = 1.2 cm). 400 μL of 1 M LiPF_6_ in propylene carbonate (Battery grade, Sigma-Aldrich) was used as liquid electrolyte. Glass fiber separator (thickness = 1.55 mm, diameter = 1.8 cm, EL-Cell GmbH) was used to separate the working electrode and counter electrode. The 2-electrode electrochemical test cell (ECC-Std, EL-Cell GmbH) was assembled in Ar atmosphere glovebox (<0.1 ppm of H_2_O and O_2_). Cyclic voltammetry of these cells was measured at a scan rate of 0.05 mV/s. Raman spectra were acquired using a Renishaw inVia confocal Raman microscope using a 532 nm laser with a power output of 22 mW. Point spectra in the LSTZ samples were acquired as 50 1-second frames and then summed to achieve high signal-to-noise ratio. No visible sample damage from the laser was found.

#### Moisture sensitivity of LSTZ0.75 ceramics and TEM specimen preparation

TEM specimens were prepared by mechanical polishing. Moisture sensitivity of LSTZ0.75 ceramics was first investigated in order to determine the material’s compatibility with polishing fluid (either water or mineral oil). Moisture sensitivity test was done by submerging the pristine LSTZ0.75 pellets in deionized water for 24 h and then dried at 100 °C for 15 min. Crystal structure, ionic conductivity, and electrochemical stability characterization of the water exposed specimen were conducted using the same method as the pristine specimen. Results and discussion of the moisture sensitivity test are reported in Supplementary Fig. 14 and in supplementary information under section titled moisture sensitivity of LSTZ0.75 ceramics.

To mechanically polish the TEM specimen, bulk pellets were first cut into rectangular blocks by a diamond saw. The rectangular blocks were 3 mm by 2 mm in area and were cut to the least possible thickness (typically ~0.5 mm) in order expedite the mechanical polishing process. Then, a single rectangular piece was placed on an Allied Multiprep Polishing System, thinned, and fine-polished against diamond lapping films (Ted Pella, Inc.) of various grit sizes. Since we have found LSTZ0.75 to be moisture insensitive, this procedure was performed with water. The thinned ceramic was glued to a molybdenum ring (Structure Probe, Inc.) with M-Bond 610 epoxy (Vishay Precision Group, Inc.). The polished pieces were then ion-milled under Ar gas for finer polishing using a Gatan model 695 Precision Ion Polishing System.

#### Microstructure characterization

STEM imaging, core-loss EELS, and SAED were conducted using a JEOL JEM 300CF operated at 300 kV. For STEM imaging, the point-to-point resolution was 0.82 Å. The microscope was equipped with double aberration correctors, Gatan Image Filter Quantum with Gatan K2 Summit and dual 100 mm^2^ Si drift detectors. Z-contrast HAADF-STEM imaging was performed with a large inner collection angle of 70 mrad, whereas BF-STEM imaging was performed with an outer collection angle of 28.4 mrad. A probe convergence semi-angle of 25.7 mrad was used for HAADF and BF-STEM imaging. EELS data were collected in STEM mode. A dispersion of 0.5 eV per channel was used to collect the edges in the ultra-high energy loss regime (Sr-L_2,3_, Zr-L_2,3_, Ta-M_2,3_, and Ta-M_4,5_ edges), while 0.1 eV per channel was used to collect the O-K edge. These EEL spectra were obtained using the Gatan K2 Summit direct detection camera. The use of a direct electron detector allows for a low electron dose and minimizes irradiation damage. Edges in the low loss regime (Sr-N_1_ and Ta-O_2,3_) were obtained using the US1000 detector, with a dispersion of 0.1 eV per channel. All core-loss EEL spectra were collected using a 2.5 mm aperture and a spectrometer collection angle of 35.89 mrad.

#### Monochromated dark field vibrational electron energy loss spectroscopy (DF VibEELS)

Vibrational EEL spectra were acquired on a Nion-UltraSTEM 200 microscope with the High Energy Resolution Monochromated EELS System (HERMES) operating at 60 kV to achieve a balance between high spatial (1.5 Å) and energy resolution (5.7 meV). Vibrational EEL spectra were collected with a 33 mrad convergence semi-angle probe and 30 mrad collection semi-angle on a CMOS detector. To exclude the contribution of non-local phonon-polaritons and maintain a strictly high spatial resolution signal, a dark-field Vibrational EELS (DF VibEELS) beam-detector geometry was employed similar to previous studies^52,56^ (Supplementary Fig. 12c). Post specimen lenses deflect the scattered beam by 65 mrad such that the bright field disk is prevented from entering the EELS collection aperture. DF VibEELS map of a (010) faceted GB with dimensions of 80 × 16 pixels was acquired with an exposure time of 2 s per pixel and a step size of 1.25 Å translating to real space dimensions of 10 × 2 nm^2^ (Supplementary Fig. 12a). Mapping of a general GB with dimensions of 80 × 10 pixels was acquired with an exposure time of 2 s per pixel and a step size of 1 Å translating to real space dimensions of 8 × 1 nm^2^ (Supplementary Fig. 12b). The contour maps in Fig. 5d, h are formed by vertically integrating the 80 × 16 and 80 × 10 pixel maps, respectively, producing 80 × 1 pixel linescans in order to improve the signal-to-noise ratio of single spectra phonon signals. The integrated spectra are then stacked horizontally side by side to produce a contour mapping with a vertical energy axis and a horizontal position axis which corresponds to the horizontal distance in the HAADF image included above the contour plot. The orange, green, and purple curves in Fig. 5b, f are constructed by integrating intensities from 10–25 meV, 25–50 meV, and 50–100 meV, respectively, and plotting them as a function of horizontal position. All microscopy studies were performed at the Irvine Materials Research Institute (IMRI) in the University of California, Irvine.

Analysis of the vibrational EEL spectra was done using custom python code. The vibrational EELS map was vertically integrated in order to improve the signal-to-noise ratio and were subsequently normalized by ZLP intensity. To isolate the phonon signal, background subtraction using an exponentiated power law function similar to the one used in previous study was carried out (Supplementary Fig. 13c)^53^.

#### Ionic conductivity calculations

Bulk and GB ionic conductivity (*σ*_b_ and ${\sigma }_{{{{{{\rm{gb}}}}}}}$, respectively) can be calculated as follow:

1 $${\sigma }_{{{{{{\rm{b}}}}}}}=\frac{L}{{R}_{{{{{{\rm{b}}}}}}}A}$$

2 $${\sigma }_{{{{{{\rm{gb}}}}}}}=\frac{L}{{R}_{{{{{{\rm{gb}}}}}}}A}$$

where *L* is the thickness and *A* is the surface area of the pellet sample. Equivalent circuit fitting (solid black trace in Supplementary Fig. 1c) of the semicircle in the higher frequency region provides the bulk resistance, *R*_b_, while fitting of the semicircle in the lower frequency region provides the GB resistance, *R*_gb_. Finally, total ionic conductivity can be calculated by:

3 $${\sigma }_{{{{{{\rm{t}}}}}}}=\frac{{\sigma }_{{{{{{\rm{b}}}}}}}{\sigma }_{{{{{{\rm{gb}}}}}}}}{{\sigma }_{{{{{{\rm{b}}}}}}}+{\sigma }_{{{{{{\rm{gb}}}}}}}}$$

To compare the real conduction behavior of the LSTZ0.75’s GBs to that of LLTO GBs, ${\sigma }_{{{{{{\rm{gb}}}}}}}^{{{{{{\rm{spec}}}}}}}$ of LSTZ0.75 is calculated using the brick layer model^6,13,67,68^. The specific grain boundary conductivity is the average conductivity of the grain boundary and is equal to:

4 $${\sigma }_{{{{{{\rm{gb}}}}}}}^{{{{{{\rm{spec}}}}}}}=\frac{L}{{R}_{{{{{{\rm{gb}}}}}}}A}\cdot \frac{\delta }{d}={\sigma }_{{{{{{\rm{gb}}}}}}}\cdot \frac{\delta }{d}$$

where *δ* is the GB width and *d* is the average grain size of the sample. The ratio of $\frac{\delta }{d}$ can be calculated according to:

5 $$\frac{\delta }{d}=\frac{{C}_{{{{{{\rm{g}}}}}}}}{{C}_{{{{{{\rm{gb}}}}}}}}$$

where *C*_g_ is the grain capacitance and *C*_gb_ is the GB capacitance. Since the equivalent circuit model used to fit the Nyquist plot contained constant phase elements (CPE) rather than real capacitor (C), the effective capacitance was derived from the fitting results using

6 $${C=({R}^{1-n}Q)}^{\frac{1}{n}}$$

where *Q* is the pseudo capacitance CPE-T and *n* (also known as CPE-P) is the degree of non-ideality (0.7–1).

#### Computational methods

Supplementary Fig. 3 shows the workflow adopted in fitting a moment tensor potential (MTP) with active learning to study structural ordering, composition, and Li-ion diffusion at bulk and GB regions of LSTZ0.75.

#### Density functional theory (DFT) calculations

All DFT calculations were performed using the Vienna ab initio simulation package (VASP) with the projector augmented-wave (PAW) approach^69,70^. The Perdew-Burke-Ernzerhof (PBE) generalized gradient approximation (GGA)^71^ was used as the exchange-correlation functional. The PBE functional was verified in our recent work to have comparable accuracy with the optB88-vdW functional^72,73^ in terms of the lattice constants, lattice expansion, and room temperature ionic conductivities of the perovskite LLTO^50^. For LSTZ0.75, the PBE simulated lattice constants are having neglectable (<1%) errors versus the experimental lattice constants from XRD (Supplementary Table 1).

For initial structural relaxations, spin-polarized calculations were performed with a plane-wave energy cutoff of 520 eV and a k-point density of at least 64/Å^−3^, same as those used in the Materials Project (MP)^74^. The global break condition for the electronic self-consistent loop (EDIFF) was set as 10^−5^ eV, in line with our previous works^37,75–77^. A low energy ordering of LSTZ0.75 at 0 K was obtained using a stepwise enumeration approach. A 2 × 2 × 2 supercell with space group of Pm$\bar{3}$m was first generated for Li_0.5_Sr_0.375_Ta_0.75_Zr_0.25_O_3_, and all unique orderings were relaxed with DFT. A $\sqrt{2}\times \sqrt{2}\times 1$ supercell of the lowest energy ordering was then constructed (Li_8_Sr_6_Ta_12_Zr_4_O_48_) and all possible orderings with substitution of the two excess Li on A-sites with one Sr and one vacancy to make Li_6_Sr_7_Ta_12_Zr_4_O_48_ (LSTZ0.75) were relaxed. The lowest energy supercell of LSTZ0.75 at 0 K is shown in Fig. 2a. It should be noted that since this structure only serves as an initial input to AIMD and MTP MC/MD simulations, an exhaustive search for the ground state ordering is not necessary and would prove too computationally expensive.

Non-spin-polarized AIMD simulations using NVT ensemble were carried out on the relaxed supercell with a plane-wave energy cutoff of 280 eV and a minimal *Γ*-centered 1 × 1 × 1 k-mesh. A timestep of 2 fs and the Nose-Hoover thermostat were used. A similar protocol was followed as previous works^37,44,50,76^, wherein simulations were performed at three strains (0, ±0.05) and four temperatures (300 K to 1200 K with 300 K intervals). The initial structures were heated from 0 K to the target temperatures with a temperature gradient of 0.25 K/fs. Snapshots were extracted from a production run of 5 ps at 0.1 ps intervals, i.e., 50 structures for each temperature and strain, resulting in a total of 600 training structures (50 × 4 temperatures × 3 strains). Static self-consistent calculations were performed on the training structures to obtain accurate energies, forces, and stresses for MTP training. These calculations were performed with a higher K-point density of at least 100/Å^−3^ and a plane-wave energy cutoff of 520 eV which were consistent with those used in MP^7^. The EDIFF was set as 10^−5^ eV, same as the relaxation calculations.

All DFT and AIMD simulations and post analysis were carried out using fully automated workflows^34^ built on the Python Materials Genomics (pymatgen) library^78^ and the FireWorks scientific workflow package^79^.

#### MTP and active learning

The MTP formalism^38,39,80,81^ has been extensively discussed in earlier works and successfully applied to many chemical systems, including metals^37,38,82^, boron^83^, alloys^80,84^, gas-phase reactions^85^, cathode coating materials^86^, and superionic conductors^50,87^. Briefly, the MTP describes the local environment around each atom in terms of moment tensors ${M}_{\mu,v}$, defined as follows:

7 $${M}_{\mu,\, v}({{{{{{\boldsymbol{n}}}}}}}_{{{{{{\boldsymbol{i}}}}}}})=\mathop {\sum }\limits_{j} \, {f}_{\mu }(|{{{{{{\boldsymbol{r}}}}}}}_{{{{{{\boldsymbol{ij}}}}}}}|,{z}_{i},{z}_{j})\underbrace{{{{{{{\boldsymbol{r}}}}}}}_{{{{{{\boldsymbol{ij}}}}}}}\otimes \cdots \otimes {{{{{{\boldsymbol{r}}}}}}}_{{{{{{\boldsymbol{ij}}}}}}}}_{\nu \,times}$$

Here, *n*_*i*_ denotes the atomic types as well as the relative positions of the *i*th atom and all its neighboring atoms. *z*_*i*_ and *z*_*j*_ represent the atomic types (integers from 0 to *n*−1 for a system with *n* different types of atoms) of the *i*th atom and its *j*th neighbor, respectively, and *r*_*ij*_ is the position vector of the *j*th neighbor to the *i*th atom. The radial part of the atomic environment is given by the *f*_*μ*_ term, and the angular part is encoded by the outer product (⨂) of the *r*_*ij*_ vectors, which is a tensor with rank *ν*. MTP then contracts the moment tensors *M*_*μ,v*_ to basis functions and applies regression to relate the energies, forces, and stresses to the basis functions.

In this work, the active learning scheme proposed by Podryabinkin and Shapeev was used to develop an MTP that can accurately simulate both bulk and GB structures^39,81^. Under this scheme, an extrapolation grade *γ* is defined to evaluate the extent to which a given configuration is extrapolative with respect to those in the training set, thereby correlating the prediction error without ab initio information. An MTP is first trained with a relatively small initial training set and then used to simulate the target system with desired conditions, i.e., similar scenarios where the MTP will eventually be used. These trial simulations will be terminated if structures with *γ* over the break threshold, *γ*_*break*_ emerge, and structures with *γ* over the select threshold, *γ*_*select*_ will be added into the training set and a new MTP is fitted. This iterative loop is repeated until the trained MTP is able to complete a simulation without the emergence of structures above *γ*_*break*_.

Our initial training set contains 600 AIMD snapshots of bulk LSTZ0.75. The MTP model complexity was set with the level of max (lev_max_) and the radial basis size equal to 12 and 8, respectively, while the cutoff radius was set as 5 Å, and the relative weights of energies, forces and stresses was set as 100:1:0.1, in line with previous studies^81,82^. For the active learning simulations, four low-sigma GB models, symmetric tilt $\varSigma 5\left[100\right](0\bar{1}2)$, simple twist $\varSigma 5\left[100\right]\left(100\right)$, symmetric tilt $\varSigma 3\left[110\right]\left(1\bar{1}1\right)$, and simple twist $\varSigma 3\left[110\right]\left(110\right)$ were utilized^88^. The ordering of Li/Sr and Ta/Zr on A and B sites inside each GB model were randomly initialized. The cell dimensions and structures are shown in Supplementary Table 4 and Supplementary Fig. 15. In each stage, the active learning iterations were executed until 100-ps MD simulations at 300–1200 K (in 100 K intervals) can be completed without the emergence of structures above *γ*_*break*_. Under a “loose break” strategy, the *γ*_*break*_ and *γ*_*select*_ were set as 20 and 5, respectively, to rapidly augment the training set and improve the MD reliability. The convergence of the MTP was achieved after 36 iterations (Supplementary Fig. 16). At this stage, the GB energies *γ*_*GB*_ were converged to within 0.15 J/m^2^ of DFT values. To avoid too large a training set, a “select-add”^81^ manipulation was applied to the aggregated training set. 623 structures were selected from the 1141 accumulated training structures and added into the new empty training set.

In the second stage, starting with the MTP optimized with the simplified training set from the 1^st^ stage, MC/MD simulations were carried out from 300 to 2100 K with 300 K intervals to the four GB models. Under an “early break” strategy, the *γ*_*break*_ and *γ*_*select*_ were both set as 20 to accelerate the convergence of active learning iterations while keeping a reasonable size of training set. For the MC runs, a swap attempt was evaluated by the Metropolis criterion at every timestep, and all possible atom swaps, i.e., the ten distinguishable combinations of atom pairs in LSTZ0.75 were considered. For the MD runs, the NPT ensembles were applied at zero pressure. The timestep was set as 1 fs. As in Supplementary Fig. 17, 100% MD reliabilities, i.e., completion of 50 ps without breaking the *γ*_*break*_ were achieved at or below 1800 K after 21 iterations of active learning, and the *γ*_*GB*_ of the four models simulated by MTPs converged very well to the DFT simulated result with an absolute error of below 0.10 J/m^2^. More importantly, this MTP was verified to reproduce DFT simulated *γ*_*GB*_ of three GB orientations out of the training set with absolute error around or below 0.10 J/m^2^ (See Supplementary Table 4). The mean absolute error (MAE) of training energies, forces, and stresses were 3.53 meV/atom, 0.20 eV/Å, and 1.84 meV/Å^3^, respectively, while the MAE of a training set of 166 GB structures extracted along MC/MD simulations were 3.21 meV/atom, 0.20 eV/Å, and 1.23 meV/Å^3^, respectively, which were comparable to previous works^37,44,50,75–77,84^. The parity plots are shown in Supplementary Fig. 18. The MTP can also accurately reproduce the DFT simulated geometrical properties, i.e., lattice constants, lattice angles, Ta/ZrO_6_ octahedron bonds, and angles with minimal errors (see Supplementary Tables 1, 2).

All training, active learning, evaluations, and simulations with MTP were performed using MLIP^38,81^, LAMMPS^89^, and the Materials Machine Learning (maml) Python package.

#### Large scale and longtime MD simulations

The DFT relaxed supercell of bulk LSTZ0.75 (77 atoms) was further enlarged by 2 × 2 × 2 and contained 616 atoms so that all lattice parameters were larger than 15 Å. MC/MD simulations were performed for 5 ns using MTP at 298, 723, 1148, and 1573 K. The MC/MD settings were the same with those in the 2^nd^ stage of active learning.

The GB models were also constructed with expanded geometrical dimensions. The interfacial distances between the two GB cores in each GB model were over 40 Å. The GB models and their dimensions are shown in Fig. 4a and Supplementary Table 3, respectively. MC/MD simulations were performed at the experimental calcination temperature of 1573 K, with the same settings as those used in the 2^nd^ stage of active learning.

Finally, MD simulations were performed to both bulk and GB models to study Li-ion diffusion in LSTZ0.75. The simulations were performed at 300 to 1000 K with 50 and 100 K intervals below and above 500 K, respectively. To collect sufficient Li-ion diffusion at lower temperatures, the simulation time was set as 10 ns and 2 ns below and above 400 K, respectively. At each temperature, five parallel MD runs were performed with randomly generated initial speeds, to average out the effect of different initial states. The NPT ensembles were used, and the timestep was set as 1 fs.

The tracer diffusivity (D*) of Li^+^ ions was obtained by performing a linear fitting of the mean square displacement (MSD), $\frac{1}{N}{\sum }_{i=1}^{N}{\left[\triangle {r}_{i}(t)\right]}^{2}$, of Li^+^ ions with time, according to the Einstein relation^90^:

8 $${D}^{*}=\frac{1}{6{Nt}}\mathop{\sum }\limits_{i=1}^{N}{\left[\triangle {r}_{i}(t)\right]}^{2}$$

where *N* is the total number of mobile species, i.e., the Li^+^ ions, and $\triangle {r}_{i}(t)$ is the displacement of the *i*th Li^+^ ion at time *t*. For the calculation of D* of at the bulk regions (${D}_{b}^{*}$), the displacements of all Li atoms inside bulk models contributed to the MSD. While for the calculation of D* of Li^+^ diffusion at the GB regions (${D}_{{gb}}^{*}$), only Li hopping inside the defined GB regions, i.e., both the start and end positions of a Li displacement within the GB region defined by GB thickness were counted in the calculation of regional MSD^51,91,92^.

The ionic conductivity (*σ*(*T*)) at temperature *T* is given by the Nernst-Einstein equation:

9 $$\sigma \left(T\right)=\frac{\rho {z}^{2}{F}^{2}}{{RT}}\cdot \frac{{D}^{*}(T)}{{H}_{R}}$$

where *ρ* is the molar density of diffusing ions in the unit cell, *z*, *F,* and *R* are the charge of Li^+^ ions (*z* = 1), the Faraday constant and the gas constant, respectively. *H*_*R*_ is the haven ratio, and in line with previous studies^51,92–95^, it is set as unity in this study, so that D* equals to the charge diffusivity (*D*_*σ*_) and will be referred as *D* from here on. Arrhenius plots were then generated to determine the temperature-dependent activation energies (*E*_*a*_).

All MC/MD and MD simulations were carried out with MLIP and LAMMPS. The visualization of bulk and GB models were conducted using OVITO^96^. The analysis on MD trajectories to extract bulk diffusivities were performed with the pymatgen-analysis-diffusion package^34,78^, and the scripts developed in this work for regional diffusivities at GB have been implemented to the same package.

#### Phonon density of states (PDOS) simulation

Force constants were calculated with the fitted MTP by the finite displacement method using a 3 × 2 × 2 supercell of the ground-state unit cell of LSTZ0.75, i.e., a supercell containing 924 atoms and lattice constants all larger than 20 Å. Under the harmonic approximation, the PDOS of LSTZ0.75 was solved and projected to each element in the structure. The absence of imaginary frequencies suggested the dynamical stability of the ground-state structure of LSTZ0.75. The generation of structures with displaced atoms and the analysis of force constants were conducted using Phonopy^97^.

## Supplementary information

Supplementary Information

## Data Availability

The data that support the findings of this study are available within the article and its Supplementary Information. The source data are available from the corresponding authors upon request.

## Code availability

The simulation code that supports the plots of this manuscript and other findings of this study are available from the corresponding authors upon request.
