# Supplementary material for: Atomic-scale origin of the low grain-boundary resistance in perovskite solid electrolyte Li0.375Sr0.4375Ta0.75Zr0.25O3
Source: Nat Commun. 2023 Apr 6;14:1940. doi: 10.1038/s41467-023-37115-6 (PMC10079928; doi:10.1038/s41467-023-37115-6)
Supplement: Supplementary file 1 — Supplementary Information [file 41467_2023_37115_MOESM1_ESM.pdf]

Supplementary Information for

Atomic-scale origin of the low grain-boundary resistance in perovskite solid electrolyte $Li_{0.375}Sr_{0.4375}Ta_{0.75}Zr_{0.25}O_3$

Tom Lee[1,6], Ji Qi[2,6], Chaitanya A. Gadre[3,6], Huaixun Huyan[1], Shu-Ting Ko[2], Yunxing Zuo[4], Chaojie Du[1], Jie Li[3], Toshihiro Aoki[5], Ruqian Wu[3*], Jian Luo[2,4*], Shyue Ping Ong[4*], and Xiaoqing Pan[1,3,5*]

[1]Department of Materials Science and Engineering, University of California at Irvine, Irvine, CA, USA.

[2]Materials Science and Engineering Program, University of California San Diego, La Jolla, CA, USA.

[3]Department of Physics and Astronomy, University of California at Irvine, Irvine, CA, USA.

[4]Department of NanoEngineering, University of California San Diego, La Jolla, CA, USA.

[5]Irvine Materials Research Institute, University of California at Irvine, Irvine, CA, USA.

[6]These authors contributed equally: Tom Lee, Ji Qi, and Chaitanya A. Gadre.

Email: xiaoqinp@uci.edu; ongsp@eng.ucsd.edu; jluo@alum.mit.edu; wur@uci.edu

Crystal structure, ionic conductivity, and electrochemical stability

Bulk $SrZrO_3$ (SZO) and LSTZ0.75 were synthesized via conventional solid state reaction method. SZO is the base material for LSTZ0.75 and is a perovskite oxide with Sr^{2+} occupying the A-sites and Zr^{4+} occupying the B-sites. For LSTZ0.75, Sr^{2+} is partially substituted by Li^{+} while Zr^{4+} is partially substituted by Ta^{5+} and displays a cubic crystal structure in the $Pm\bar{3}m$ space group[1]. Supplementary Fig. 1a displays two unit cells of the proposed crystal structure of LSTZ0.75. Supplementary Fig. 1b shows X-ray diffraction (XRD) pattern of SZO and LSTZ0.75 where the diffraction peaks shifted toward higher 2θ with increasing Li and Ta contents from undoped SZO (y = 0) to LSTZ0.75. The ionic radii of dopant elements Li^{+} (76 pm) and Ta^{5+} (64 pm) are smaller than those of Sr^{2+} (144 pm) and Zr^{4+} (72 pm)[2], respectively, which would result in a decrease in lattice parameters a as both Li^{+} and Ta^{5+} dopant content increase. The increase in 2θ indicates a decrease in lattice parameters, so it is expected that Li^{+} and Ta^{5+} are successfully substituting Sr^{2+} and Zr^{4+}, respectively, in LSTZ0.75. Lattice parameters a of SZO and LSTZ0.75 are estimated to be 4.11 Å and 4.037 Å, respectively, which are consistent with the values reported in literature[3,4]. The ionic conductivity and electrochemical stability of LSTZ0.75 was investigated by AC impedance spectroscopy and cyclic voltammetry (CV), respectively. Supplementary Fig. 1c shows the Nyquist plot (dotted blue trace) of LSTZ0.75 measured at 25 °C. 2 semicircles were obtained in high-frequency region, while the linear tails were obtained at low-frequency region, indicating that the conduction nature is primarily ionic. Equations for calculating the ionic conductivities are shown in the Methods section. The calculated bulk, GB, and total ionic conductivities (σ_{b}, σ_{gb}, and σ_{t}, respectively) of LSTZ0.75 are σ_{b} = 2.32 × 10^{-4} S/cm, σ_{gb} = 1.34 × 10^{-3} S/cm, and σ_{t} = 1.98 × 10^{-4} S/cm, which are consistent with reports in literature[5]. It is worth noting that the σ_{gb} is much higher than σ_{b}, contrary to that in perovskite-type LLTO, where σ_{gb} is much lower than σ_{b}. To compare the real conduction behavior of the LSTZ0.75's GBs to that of LLTO GBs, $\sigma_{\mathrm{gb}}^{\mathrm{spec}}$ of LSTZ0.75 is calculated using the brick layer model[6–9]. From the fitting results, we calculated $\sigma_{\mathrm{gb}}^{\mathrm{spec}}$ = 7.96 × 10^{-7} S/cm for LSTZ0.75, which is significantly higher than (~26× of) $\sigma_{\mathrm{gb}}^{\mathrm{spec}}$ = 3.1 × 10^{-8} S/cm of LLTO[6]. This indicates lithium-ion transport in the GBs of LSTZ is significantly faster than that in GBs of LLTO. To understand mechanism behind the improved lithium-ion conduction, GB microstructure of LSTZ0.75 needs to be studied. Supplementary Fig. 1d displays the first cycle CV of LSTZ0.75 measured at 0.05 mV/ s. A reduction peak at 0.74 V *vs.* Li/Li^{+} and an onset insertion potential of ~0.91 V vs. Li/Li^{+} are

observed, which is consistent with previous study[1]. Results from crystal structure, ionic conductivity, and electrochemical stability characterization demonstrate that our as-synthesized LSTZ0.75 ceramics have bulk properties similar to those reported in literature.

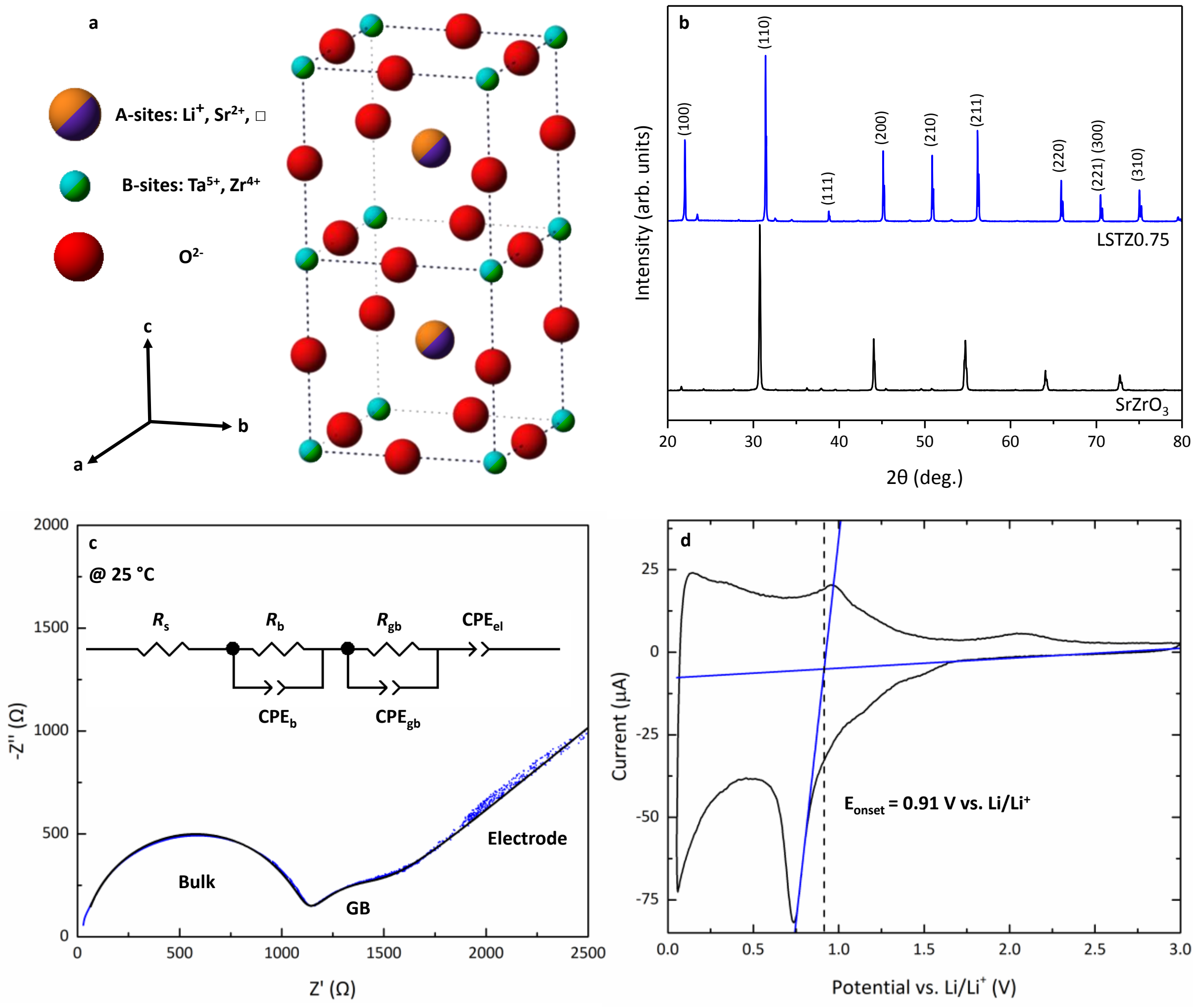

Supplementary Fig. 1 | Schematic and general characterization of pristine LSTZ0.75. a, Crystal structure of cubic LSTZ0.75. **b,** X-ray diffraction patterns of $SrZrO_3$ and LSTZ0.75. **c,** AC impedance spectra of LSTZ0.75 measured at 25 °C. The measured raw data is plotted in blue dotted line, while the fitted curve is plotted in black solid line. Equivalent circuit model used to fit the data is shown in **c**. **d** First cycle cyclic voltammogram of LSTZ0.75/ Li cell, measured at a scan rate of 0.05 mV/ s.

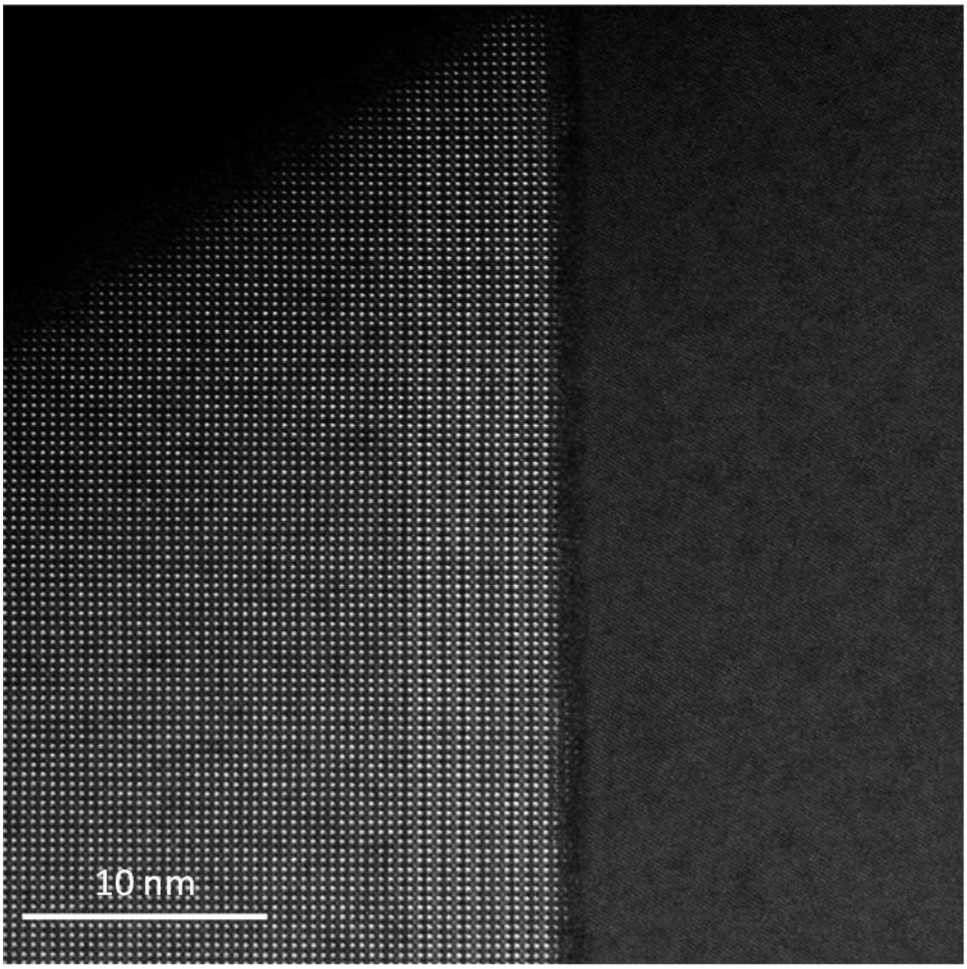

Supplementary Fig. 2 | Numerous dark spots observed in the grain interior of both grains. Low magnification atomic-resolution HAADF-STEM image of the (010) faceted GB shown in Fig. 2a.

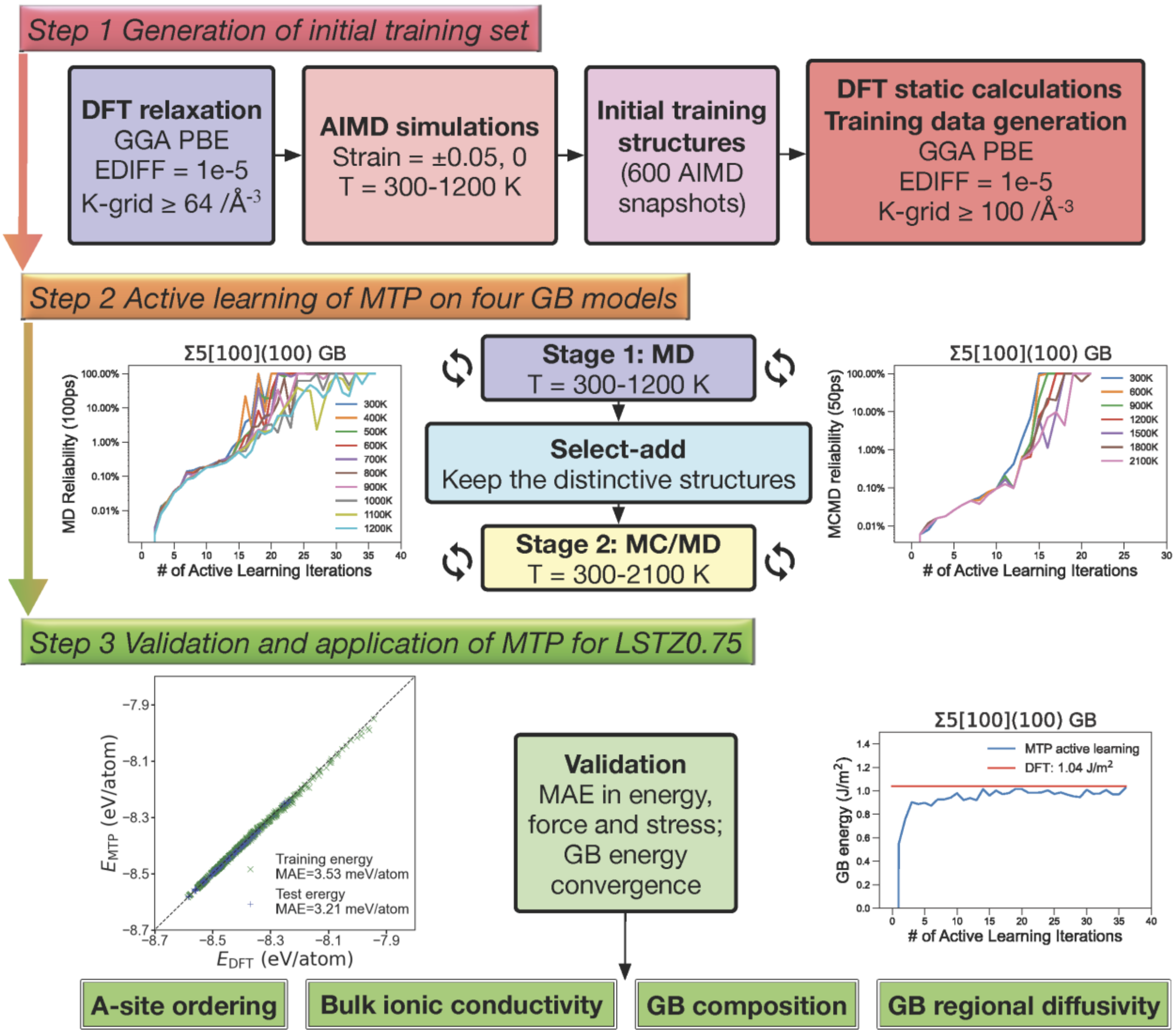

Supplementary Fig. 3 | Active learning workflow to fit an MTP for both bulk and GB structures of LSTZ0.75.

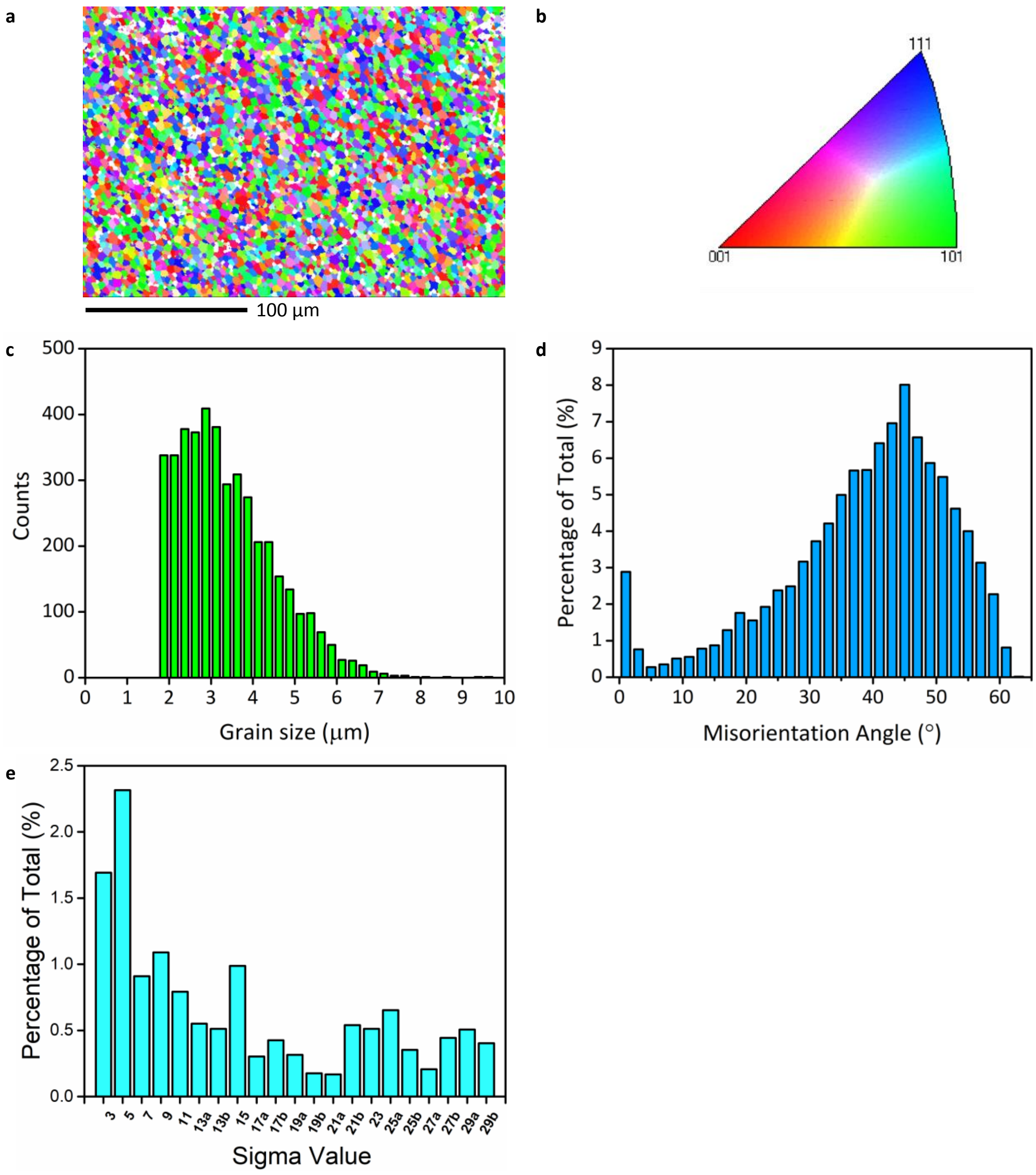

Supplementary Fig. 4 | Electron Back Scattering Diffraction (EBSD) shows that the average grain size of LSTZ0.75 is 3.38 ± 1.13 μm, and most of the GBs are composed of grains with random orientations and the concentration of small-angle grain boundaries are very small. a, orientation map of one region in a LSTZ0.75 pellet and **b,** its corresponding IPF color key. Histograms of **c,** the grain size distribution, **d,** the grain misorientation angle distribution, and **e,** the GB sigma value distribution generated based on the orientation map.

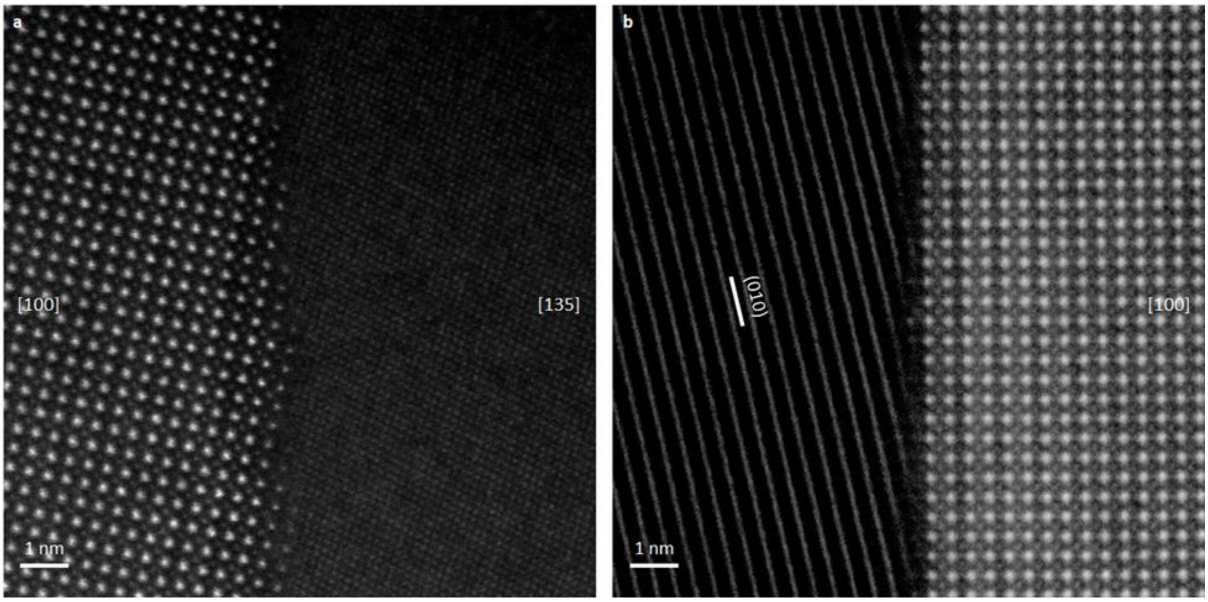

Supplementary Fig. 5 | Same crystal lattices from the grain bulk extend all the way to the GBs. High magnification atomic-resolution HAADF-STEM images of **a,** the general grain boundary from Fig. 1g and **b,** a (010) faceted grain boundary (with respect to the right-side grain). The zone axes parallel to the incident electron beam were indicated. The left-side grain in **b** was not oriented along any particular zone axis, and the crystallographic plane corresponding to the lattice fringes was indexed.

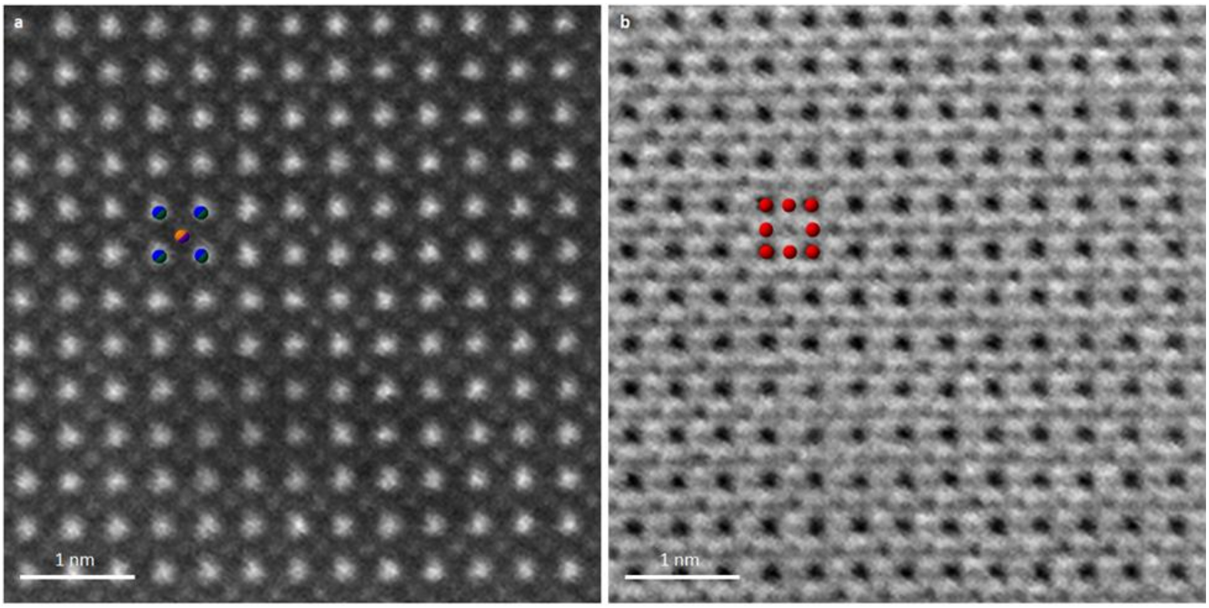

Supplementary Fig. 6 | Additional atomic-scale study of grain interior. Atomic-resolution **a,** HAADF-STEM and **b,** BF-STEM image of LSTZ0.75 grain bulk. Atoms of a single unit cell (A-site centered view) are labeled in accordance with the schematic of LSTZ0.75 crystal structure (Supplementary Fig. 1a).

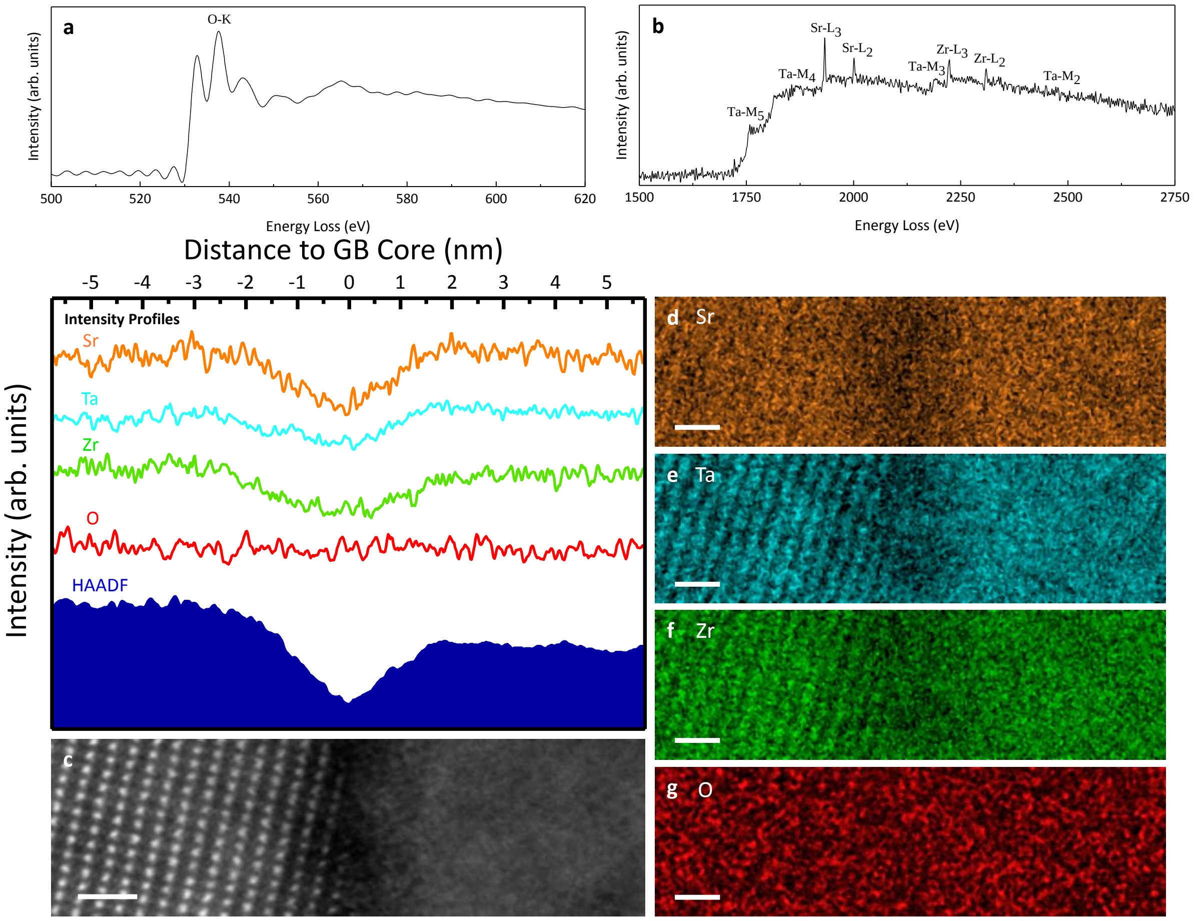

Supplementary Fig. 7 | Core-loss EELS data of a general GB. Integrated EEL spectra of **a,** O-K and **b,** Sr-$L_{2,3}$, Zr-$L_{2,3}$, Ta-$M_{2,3}$, and Ta-$M_{4,5}$ edges for a general grain boundary shown in **c**. **c,** Atomic-resolution HAADF-STEM image of a general grain boundary. Elemental maps of **d,** Sr, **e,** Ta, **f,** Zr, and **g,** O. All scale bars are 1 nm. Intensity profiles of **c** – **g** are shown above **c**.

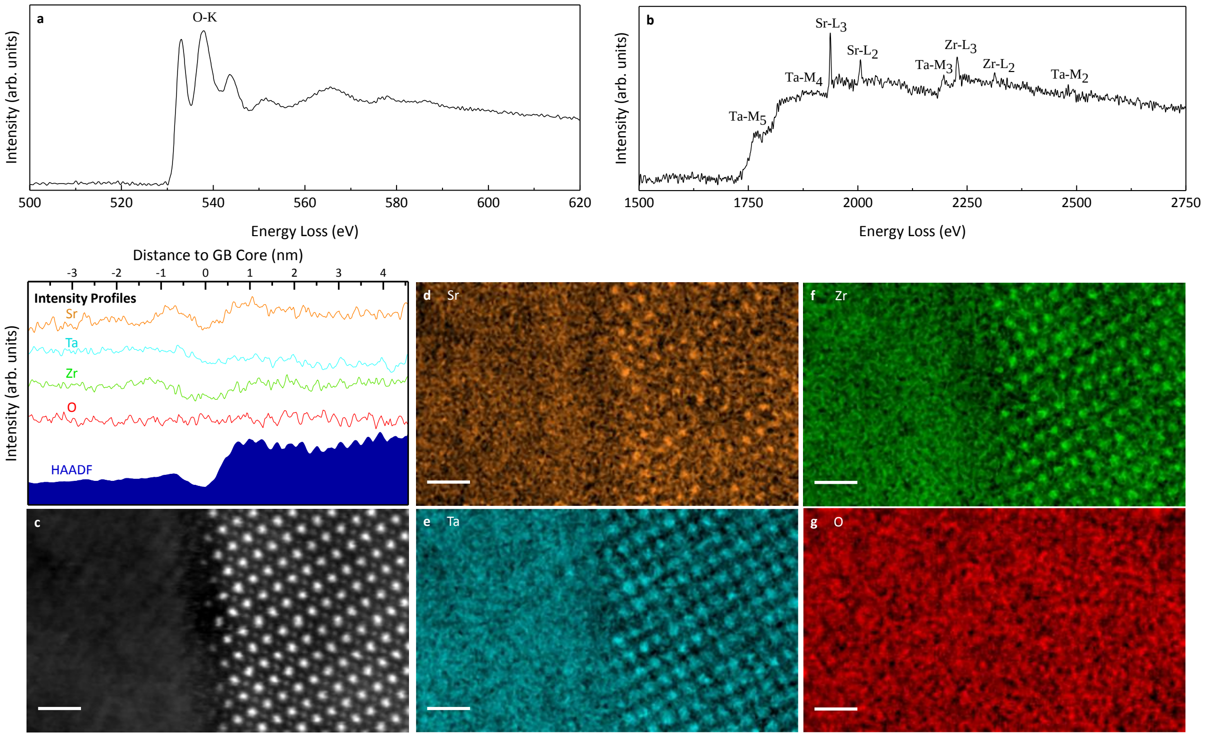

Supplementary Fig. 8 | Core-loss EELS data of another general GB. Integrated EEL spectra of **a,** O-K and **b,** Sr-$L_{2,3}$, Zr-$L_{2,3}$, Ta-$M_{2,3}$, and Ta-$M_{4,5}$ edges for a general grain boundary shown in **c**. **c,** Atomic-resolution HAADF-STEM image of a general grain boundary. Elemental maps of **d,** Sr, **e,** Ta, **f,** Zr, and **g,** O. All scale bars are 1 nm. Intensity profiles of **c** – **g** are shown above **c**.

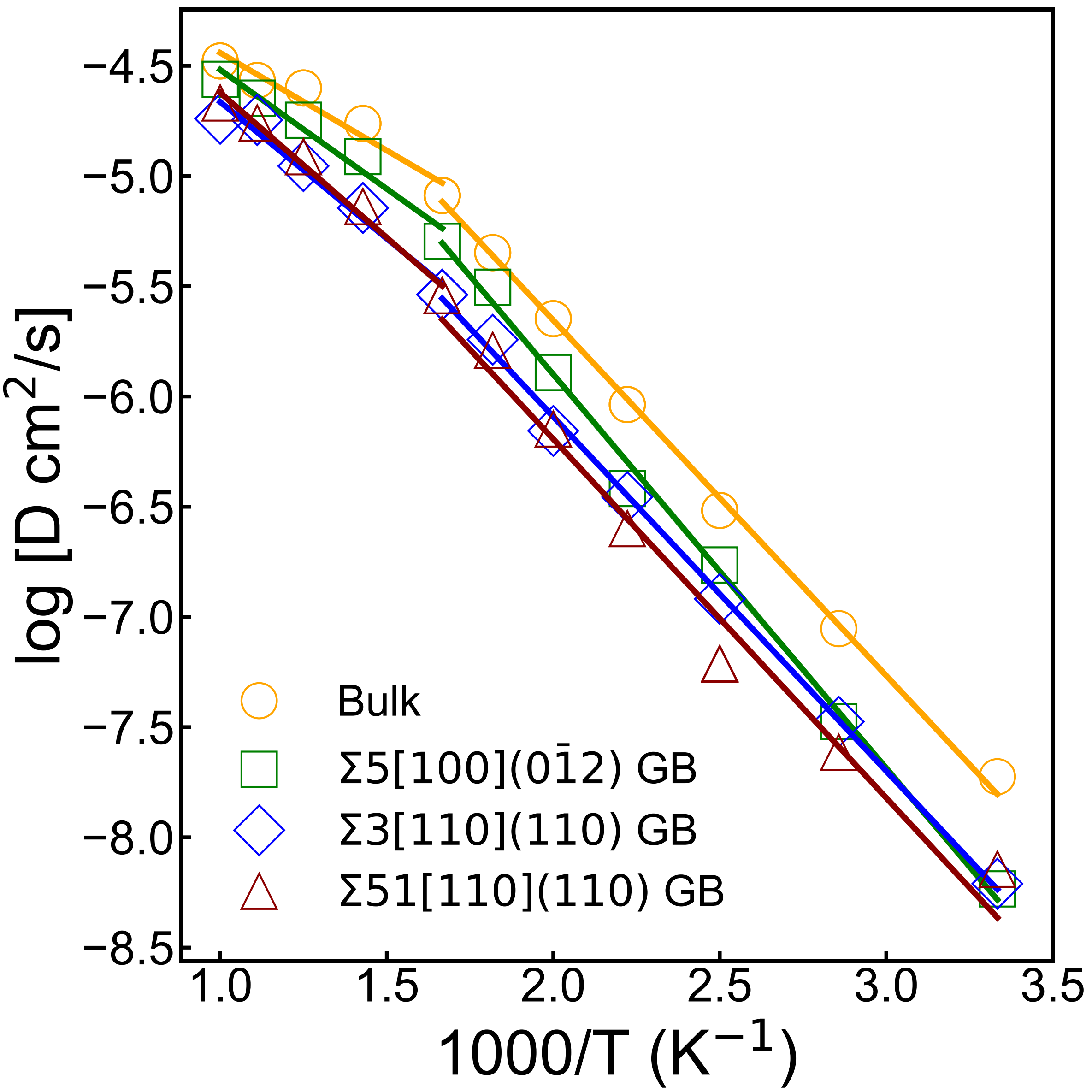

Supplementary Fig. 9 | The Arrhenius plot of Li diffusivity in LSTZ0.75. The Li diffusivities were calculated using the bulk model, the two most stable low-sigma GBs and the high-sigma GB. The GB models have not been equilibrated. The respective $D_{\text{Li, 300K}}$ and E_a were provided in Table 2. The Arrhenius plot of the equilibrated GB models was provided as Fig. 4c.

Challenge in determining Li distribution in LSTZ0.75 microstructure by conventional low energy core-loss EELS

An attempt to investigate the Li distribution at the GBs is made by conducting EELS at the low energy loss regime. Supplementary Fig. 10 shows EEL spectra of LSTZ0.75 (black curve), $SrZrO_3$ (green curve), and Ta_2O_5 (blue curve). There are two peaks in the LSTZ0.75 low loss EEL spectrum, one at 42.5 eV and another at 52.5 eV. Both peaks are too low in energy loss to be assigned as the Li-K edge, which typically appears at 55-65 eV[10,11]. Due to the lack of literature on EELS of Sr, Ta, and Zr, we conducted EELS measurements of $SrZrO_3$ and Ta_2O_5 in order to assign the two peaks found in EEL spectrum of LSTZ0.75. EEL spectrum of $SrZrO_3$ showed a single peak at 42.5 eV which is assigned to the Sr-N_1 edge. Similarly, the EEL spectrum of Ta_2O_5 showed a single peak at 52.5 eV which can be ascribed to the Ta-$O_{2,3}$ edge. Based on these finding, the peaks at 42.5 and 52.5 eV in EEL spectrum of LSTZ0.75 can be assigned as Sr-N_1 edge and Ta-$O_{2,3}$ edge, respectively. Due to the broad shape of the Ta-$O_{2,3}$ edge and its proximity to the Li-K edge, the Ta-$O_{2,3}$ edge overwhelms the Li-K edge. This is reasonable, though certainly unfortunate, as the nominal composition $Li_{0.375}Sr_{0.4375}Ta_{0.75}Zr_{0.25}O_3$ has twice as much Ta as Li. As a result, mapping of the Li distribution in grains and GBs of LSTZ0.75 via the Li-K edge is not feasible in systems which contain a large Ta:Li ratios. In fact, previous reports have found that perovskite-type solid electrolytes in general are difficult to identify the Li-K edges with certainty[11,12]. The reason is that perovskite-type solid electrolytes contain lower volume densities of Li atoms than other types of solid electrolytes.

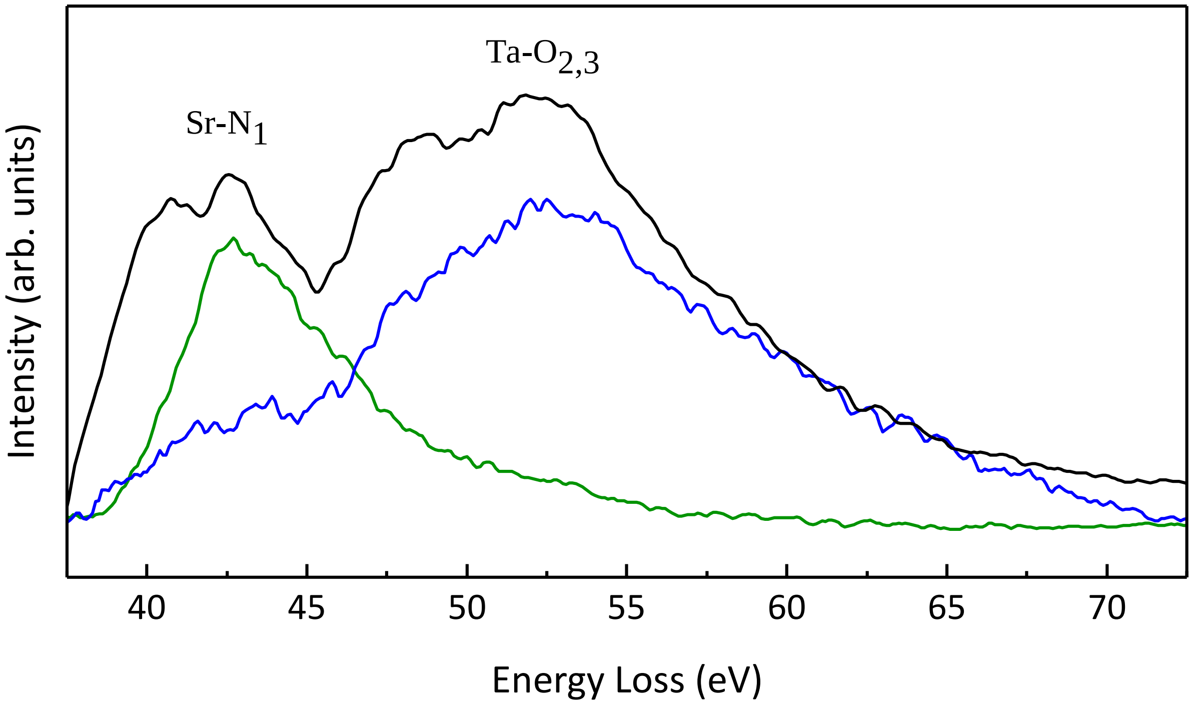

Supplementary Fig. 10 | Distinct Li-K edge of LSTZ0.75 is not observed due to its close proximity to the Ta-$O_{2,3}$ edge. Integrated EEL spectra collected at the low energy loss regime. Edge corresponding to each peak is assigned.

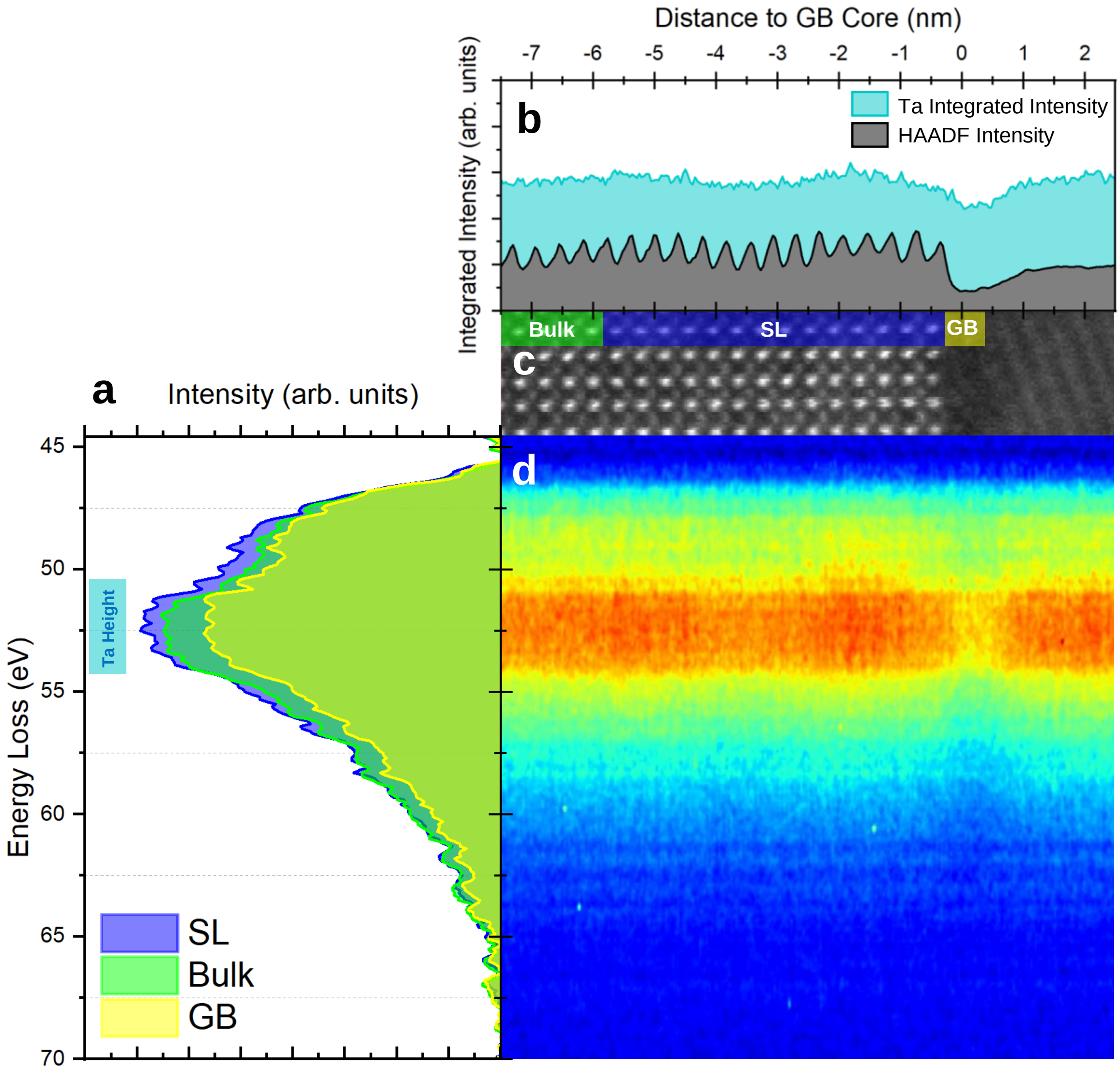

Supplementary Fig. 11 | Low-Loss EELS of (010) faceted GB. a, Representative spectra of Ta-$O_{2,3}$ edge at the SL, bulk, and GB regions. Average Ta-$O_{2,3}$ height is higher at the SL region than both the bulk and GB. The cyan-colored label above the peak denotes the energy range over which the Ta-$O_{2,3}$ height was averaged. **b**, Line profile of the averaged Ta-$O_{2,3}$ height overlaid with vertically integrated HAADF line profile. **c**, HAADF image of the region corresponding to the region low energy core-loss EELS was acquired. **d**, Contour plot of spectral slices stacked horizontally. The vertical axis denotes energy loss while the horizontal axis denotes the horizontal real-space position corresponding to the HAADF in **c**.

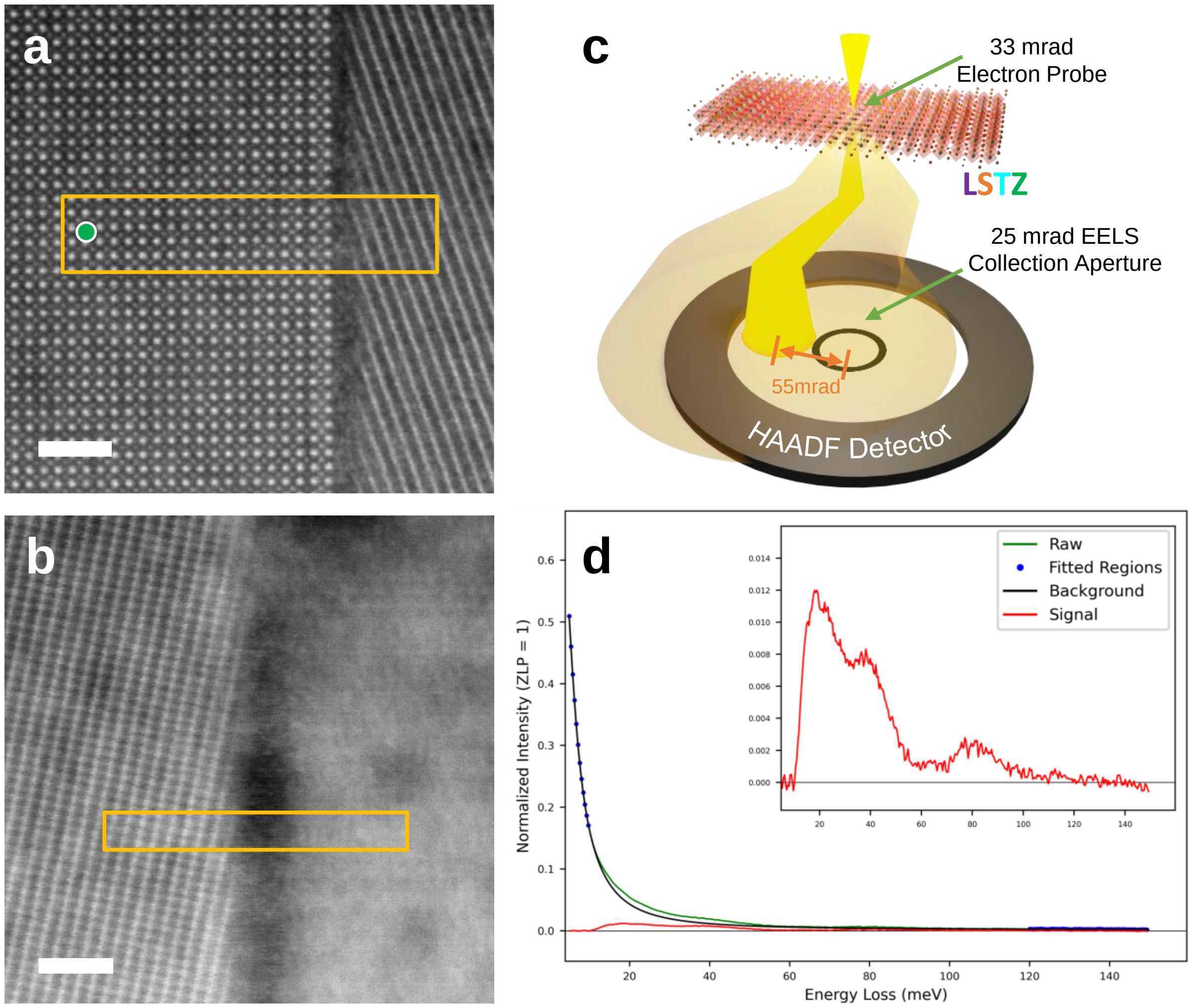

Supplementary Fig. 12 | DF VibEELS Beam-Detector Geometry and Spectra. a, HAADF image where mapping data Fig. 5a-d was obtained. **b**, HAADF image where mapping data Figs. 5e-h was obtained. The regions outlined in orange in **a** and **b** correspond to the vibrational map sizes. Scale bars denote 2 nm. **c**, DF VibEELS beam-detector geometry illustrating a deflected post-specimen scattering beam and the collection of off-axis scattered electrons producing a purely local vibrational signal. The direct beam is deflected by 55 mrad from the collection aperture center. **d**, Background subtraction of DF VibEEL spectra obtained from the region denoted by the green circle in **a**. The green colored line represents the normalized as-acquired spectra in the corresponding regions. Blue dots represent the spectrum region used for fitting the background. The red curves in the main plot and in the inset represent the background subtracted signal.

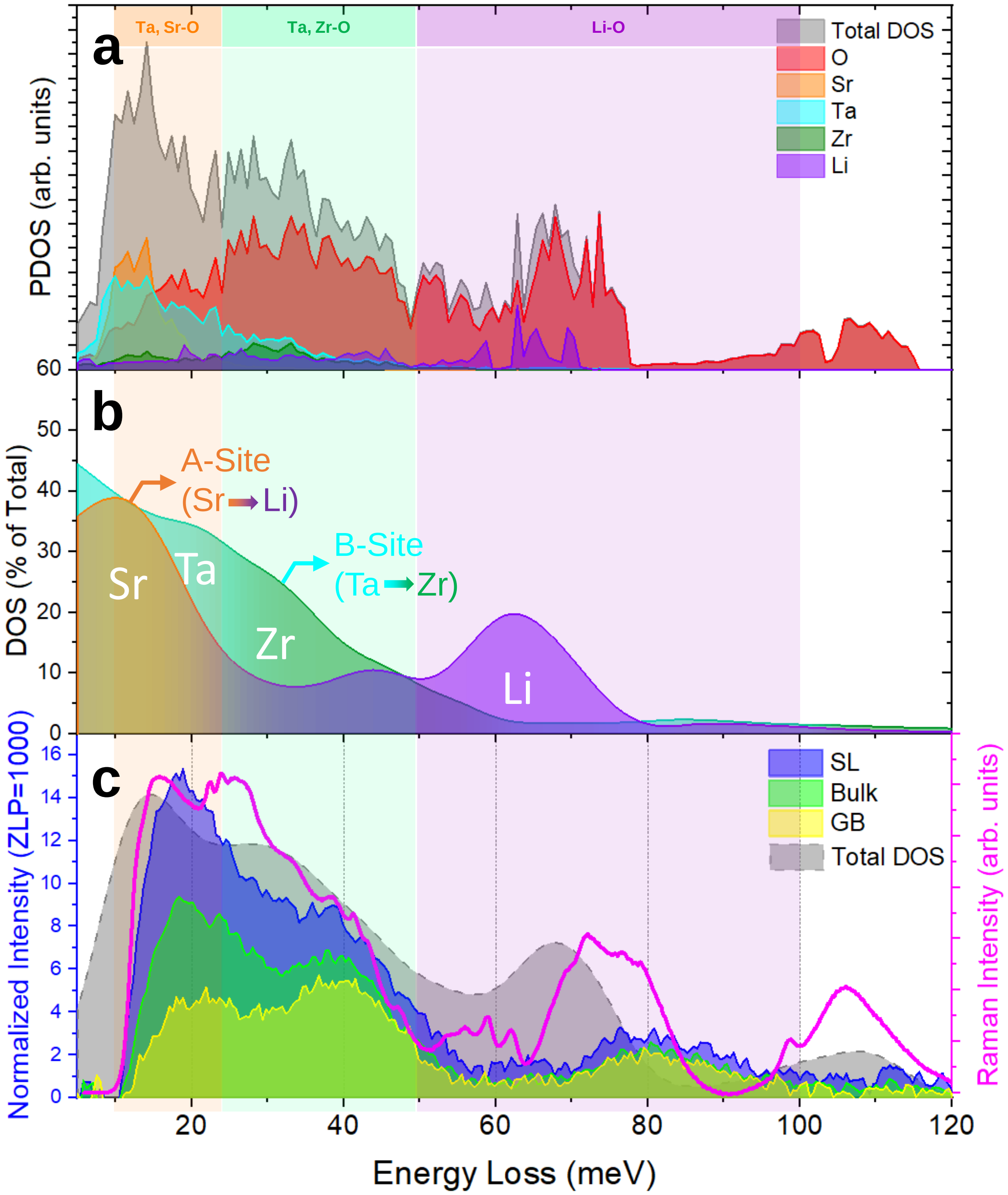

Supplementary Fig. 13 | Elemental Contributions to Total Phonon Density of States and Vibrational EELS. a, Phonon Density of States (PDOS) along with individual elemental contributions. Labels at the top indicate energy ranges where the labeled vibrations are most dominant. **b**, A, B-site contributions normalized by total PDOS convolved with a 9 meV gaussian to match experimental DF VibEELS energy resolution. A-site and B-site curves are the sum of Li and Sr, and Ta and Zr projected PDOS elemental contributions, respectively. The orange and purple, and cyan and green colors represent varying percentage of Sr and Li, and Ta and Zr, respectively, as indicated by the labels. **c**, Plots of convolved PDOS, Raman spectra of the LSTZ pellet, and DF VibEEL spectra in the SL, Bulk, and GB regions. An integration width of 50-100 meV has been chosen for Li-O vibration due to the known underestimation of energies in the PDOS.

Moisture sensitivity of LSTZ0.75 ceramics

In order to prepare TEM specimen using mechanical polishing method, we first investigate the moisture sensitivity of LSTZ0.75 ceramics. Numerous Li-ion conductors are known to be moisture sensitive and exposing them to water would result in either structural change or formation of secondary phases[13–16]. These changes would lead to a decrease in their total ionic conductivity. Therefore, cutting and mechanical polishing of these materials would have to be performed with mineral oil instead of water. However due to the lack of literature on LSTZ, its stability against water is unknown[14].

Thus, we investigated moisture sensitivity of LSTZ0.75 by examining its structural quality, ionic conductivity, and electrochemical stability after being submerged in deionized water for twenty four hours. Supplementary Fig. 12a shows the XRD diffractograms of pristine and water exposed LSTZ0.75. No difference was observed when comparing the two XRD patterns, indicating that there was no structural change or formation of secondary phases in the water exposed LSTZ0.75 sample. Supplementary Fig. 12b shows Nyquist plot of the water exposed LSTZ0.75. Using the same calculations as described in the Methods section, the values of σ_b, σ_{gb}, and σ_t at 25 °C for LSTZ0.75 after submerging 24 hours in deionized water are determined to be 2.37×10^{-4} S/cm, 9.16×10^{-4} S/cm, and 1.88×10^{-4} S/cm, respectively. Minimal change in the ionic conductivity values between the pristine and the water exposed sample is observed. Supplementary Fig. 12c displays the first cycle CV of water exposed LSTZ0.75 measured at 0.05 mV/ s. A reduction peak at 0.73 V vs. Li/Li^+ and an onset insertion potential of ~0.9 V *vs.* Li/Li^+ are observed, which indicates a similar electrochemical stability to the pristine LSTZ0.75 sample. Finally, Supplementary Fig. 12d shows the Raman spectra of pristine and water exposed LSTZ0.75. No difference is observed when comparing the two spectra, indicating there is no change in the chemical bonds after pristine LSTZ0.75 has been exposed to water. Moreover, no band at 1090 cm^{-1} corresponding to the vibration of CO_3^{2-} was observed in the Raman spectra[17,18], which means LSTZ0.75 did not react with moist air or water to form Li-insulating $LiCO_3$.

The above observations indicate that LSTZ0.75 is not moisture sensitive. This stability against water could be caused by the weak Zr^{4+} and Ta^{5+} bond with O^{2-}, due to their relatively large ionic radii. Recall that interatomic distance determines the bond strength: long interatomic distance will result in low bond strength. Since the Li–O/Sr–O and Zr–O/Ta–O bonds in LSTZ0.75 share the same O 2p orbitals, a weaker B–O bond results in a

stronger A–O bond, vice versa[19]. The strong Li–O bond prevents Li^+ from reacting with H_2O, making LSTZ0.75 not sensitive to water. This is the first-time moisture insensitivity of LSTZ has been reported. Its stability against water is advantageous for practical use as a solid-state electrolyte. Coupled with the appropriate anode and cathode, LSTZ0.75 can be used as solid electrolyte to assemble an ASSB in ambient environment. This greatly decreases production cost and allows ASSB to be competitive against convention LIB. LSTZ solid electrolyte may also have an application in lithium-air batteries based on aqueous electrolytes and in lithium-redox flow batteries in aqueous/ aprotic hybrid electrolyte system[14,17]. Practical applications that exploit LSTZ's moisture insensitivity deserves future in-depth investigation. For our present study, cutting and mechanical polishing of the LSTZ0.75 ceramics are performed with deionized water when preparing the TEM specimen.

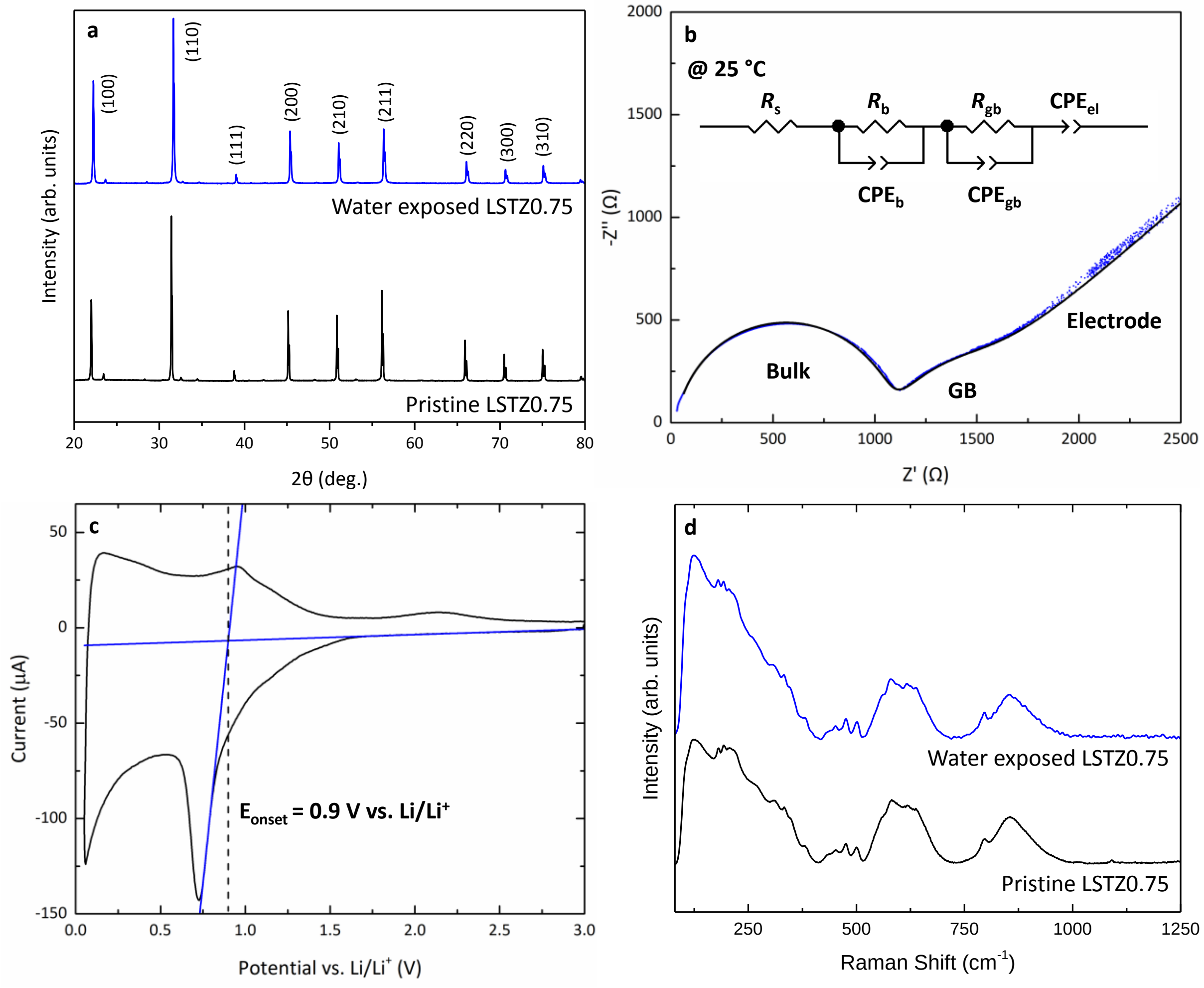

Supplementary Fig. 14 | General characterization of water exposed LSTZ0.75. a, X-ray diffraction patterns of pristine LSTZ0.75 and water exposed LSTZ0.75. **b,** AC impedance spectra of water exposed LSTZ0.75 measured at 25 °C. The measured raw data is plotted in blue dotted line, while the fitted curve is plotted in black solid line. Equivalent circuit used to fit the data is shown. **c,** First cycle cyclic voltammogram of water exposed LSTZ0.75/ Li cell, measured at a scan rate of 0.05 mV/ s.

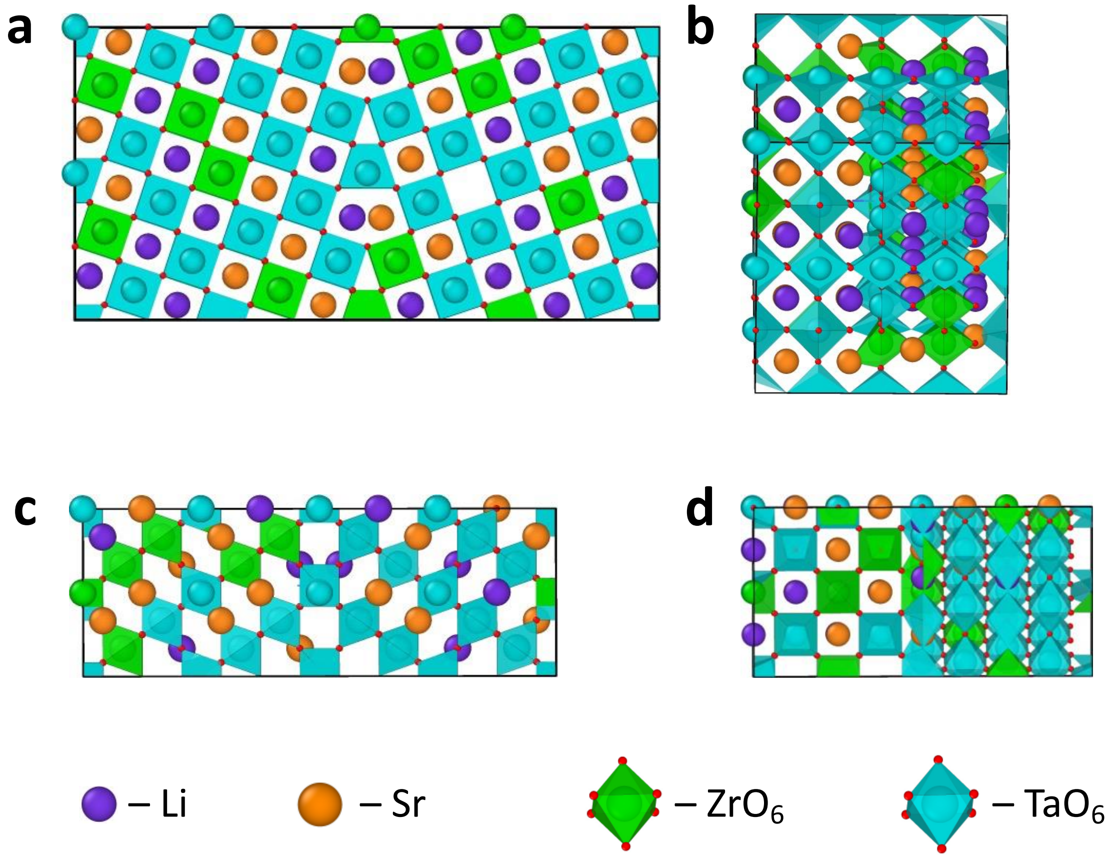

Supplementary Fig. 15 | Structures of the four low-sigma GB models of LSTZ0.75 used in the active learning of MTP. The GB orientations are **a,** symmetric tilt $\Sigma5[100](0\bar{1}2)$, **b,** Simple twist $\Sigma5[100](100)$**, c,** Symmetric tilt $\Sigma3[110](1\bar{1}1)$ and **d,** Simple twist $\Sigma3[110](110)$. The GB dimensions and GB energies by DFT and MTP were listed in Supplementary Table 4.

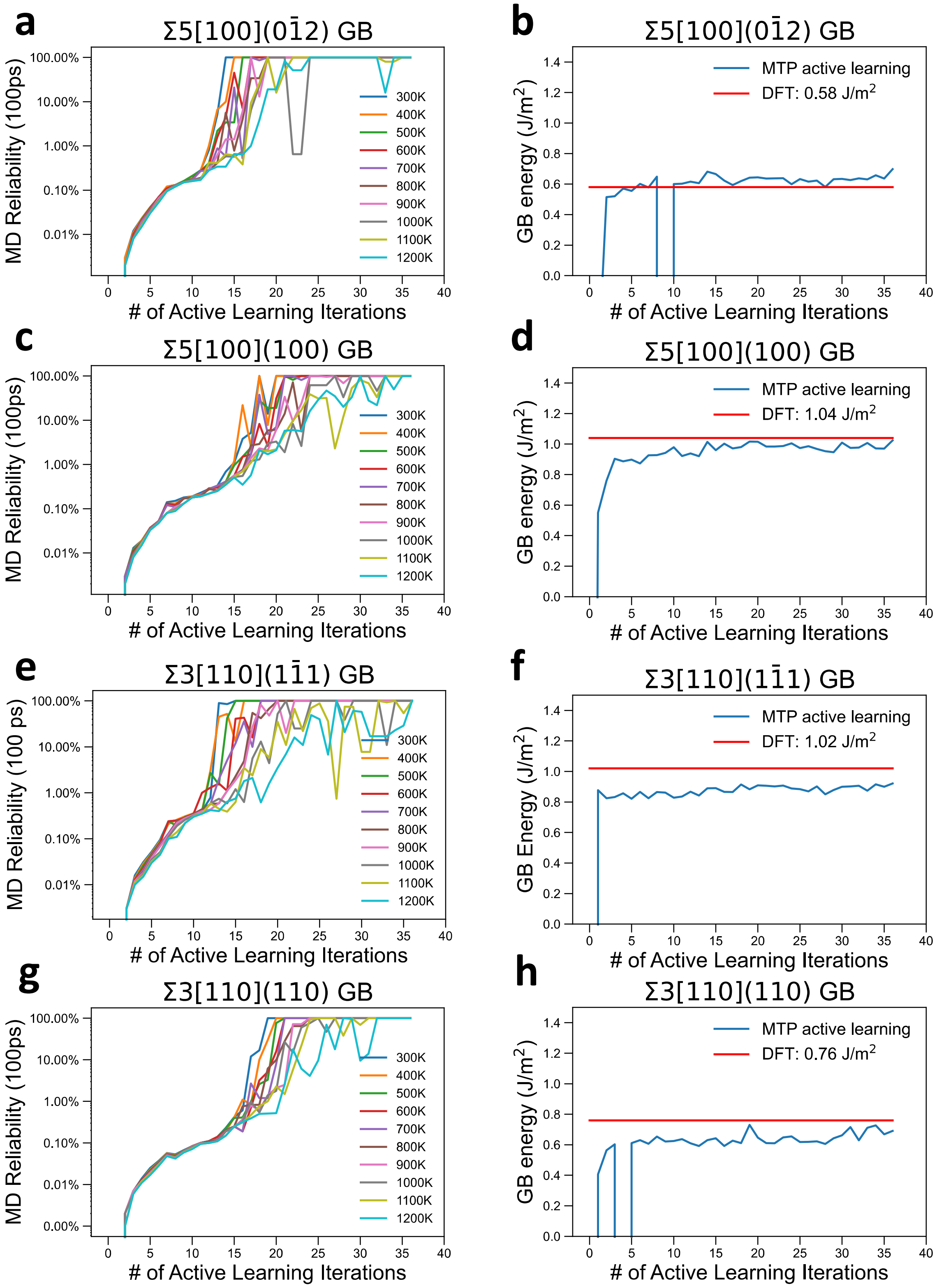

Supplementary Fig. 16 | Convergence of the stage 1 of active learning for MTP. Evolution of MD reliabilities in the stage 1 of active learning of MTP for **a,** Σ5[100]$(0\bar{1}2)$ GB, **c,** Σ5[100](100) GB, **e,** Σ3[110]$(1\bar{1}1)$ GB, and **g,** Σ3[110](110) GB. Convergence of GB energies in the stage 1 of active learning of MTP for **b,** Σ5[100]$(0\bar{1}2)$ GB, **d,** Σ5[100](100) GB, **f,** Σ3[110]$(1\bar{1}1)$ GB, and **h,** Σ3[110](110) GB.

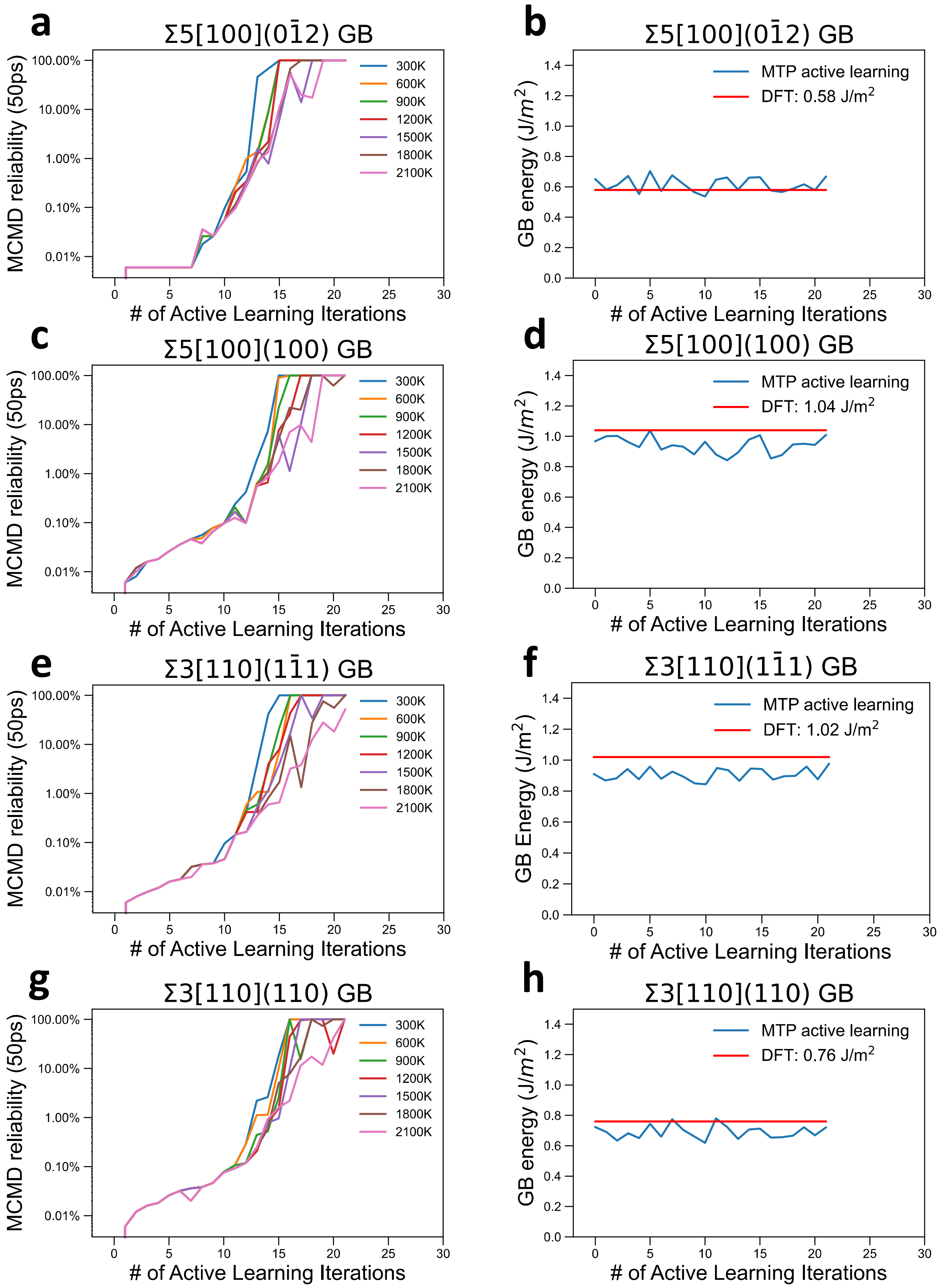

Supplementary Fig. 17 | Convergence of the stage 2 of active learning for MTP. Evolution of MC/MD reliabilities in the stage 2 of active learning of MTP for **a,** $\Sigma5[100](0\bar{1}2)$ GB, **c,** $\Sigma5[100](100)$ GB, **e,** $\Sigma3[110](1\bar{1}1)$ GB, and **g,** $\Sigma3[110](110)$ GB. Convergence of GB energies in the stage 2 of active learning of MTP for **b,** $\Sigma5[100](0\bar{1}2)$ GB, **d,** $\Sigma5[100](100)$ GB, **f,** $\Sigma3[110](1\bar{1}1)$ GB, and **h,** $\Sigma3[110](110)$ GB.

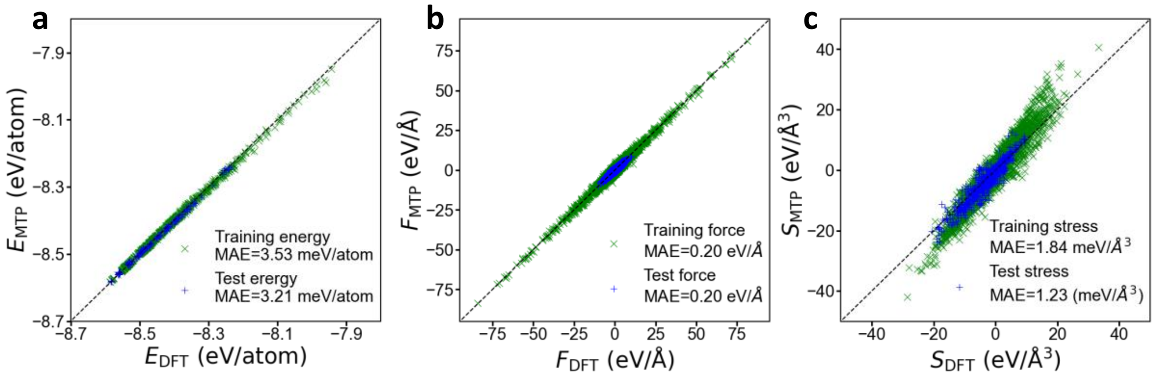

Supplementary Fig. 18 | Error distribution of the training and test errors. The parity plots for the error distribution of training and test **a,** energies, **b,** forces and **c,** stresses of the optimized MTP after two stages of active learning iterations. The training set contains 864 structures generated and selected in the utilized workflow (Supplementary Fig. 3). The test set has 166 structures extracted from 1 ns MC/MD simulations of the four low-sigma GB models at 298 to 1573 K with 425 K intervals by the trained MTP.

Supplementary Table 1 | Lattice parameters and lattice angles predicted by DFT and MTP relaxations at 0 K. Percentage errors versus experimental lattice constant by analyzing XRD results with Bragg's law are provided.

	Lattice constants (Å)			Lattice angles (º)		
	a	b	c	α	β	γ
Expt.	4.037	4.037	4.037	90	90	90
DFT	4.03(-0.2%)	4.044(0.2%)	4.044(0.2%)	89.8(-0.3%)	89.9(-0.1%)	90.1(0.1%)
MTP	4.034(-0.1%)	4.041(0.1%)	4.041(0.1%)	90(0%)	90(0%)	90(0%)

Supplementary Table 2 | Averaged bond angles (∠) and distances (d) of TaO_6 and ZrO_6 octahedrons predicted by DFT and MTP relaxation at 0 K. The standard deviations of the averaged values were provided in brackets.

	TaO_6 octahedrons		ZrO_6 octahedrons	
	$\angle_{O\text{-}Ta\text{-}O}$ (º)	$d_{Ta\text{-}O}$ (Å)	$\angle_{O\text{-}Zr\text{-}O}$ (º)	$d_{Zr\text{-}O}$ (Å)
DFT	89.80 (5.54)	2.01 (0.08)	89.77 (6.40)	2.11 (0.06)
MTP	89.84 (5.04)	2.01 (0.07)	89.80 (5.87)	2.11 (0.05)

Supplementary Table 3 | Interplanar distance ($d_{interplanar}$), GB thickness (d_{GB}), cell dimensions (x, y and z; z is perpendicular to GB planes) and total number of atoms (N_{total}) of GB models of LSTZ0.75. The d_{GB} was defined to be either two or four times the $d_{interplanar}$ of GB planes in the lattice with a general requirement of d_{GB} > 5 Å.

GB orientations	$d_{interplanar}$ (Å)	GB angle (º)	d_{GB} (Å)	GB dimensions (Å)			N_{total}
				x	y	z	
$\Sigma5[100](0\bar{1}2)$	1.79	53.13	7.14	15.97	17.85	89.26	1925
$\Sigma5[100](100)$	3.99	53.13	7.98	17.85	17.85	87.82	2117
$\Sigma3[110](1\bar{1}1)$	2.30	109.47	9.22	16.94	16.94	82.97	1560
$\Sigma3[110](110)$	2.82	109.47	5.65	20.74	19.56	90.33	2772
$\Sigma51[110](100)$	3.99	22.84	7.98	28.51	40.32	90.33	7854

Supplementary Table 4 | Cell dimensions (x, y and z; z is perpendicular to GB planes) and total number of atoms (N_{total}) of GB models of LSTZ0.75 used in the active learning iterations as well as in the verification of MTP. GB energy (γ_{GB}) calculated by DFT and by the fitted MTP for each GB orientation were provided.

GB orientations	GB dimensions (Å)			N_{total}	γ_{GB} (J/m^2)	
	x	y	z		DFT	MTP
GB orientations used in the active learning iterations of MTP						
$\Sigma5[100](0\bar{1}2)$	7.98	17.85	35.71	385	0.58	0.67
$\Sigma5[100](100)$	17.85	17.85	15.97	385	1.04	1.01
$\Sigma3[110](1\bar{1}1)$	11.29	11.29	27.66	231	1.02	0.98
$\Sigma3[110](110)$	11.98	11.98	22.58	231	0.76	0.72
GB orientations out of training set and used in the verification of MTP						
$\Sigma3[110](1\bar{1}2)$	11.29	13.83	19.56	231	0.63	0.74
$\Sigma3[110](001)$	23.95	17.85	23.95	693	1.43	1.43
$\Sigma9[110](110)$	23.95	33.87	11.29	693	0.99	1.05

Supplementary Table 5 | Anti-site energies of all possible atom swaps in the $\Sigma3[110](110)$ GB predicted by DFT.

Atom swap	Anti-site energy (eV)			
	GB ↔ bulk	bulk ↔ GB	GB ↔ GB	bulk ↔ bulk
Li ↔ Sr	5.0	3.2	4.7	3.5
Li ↔ Ta	17.9	16.4	16.0	16.2
Li ↔ Zr	12.3	7.3	9.8	11.0
Li ↔ O	20.2	20.8	20.7	19.7
Sr ↔ Ta	22.7	20.7	21.4	21.9
Sr ↔ Zr	12.6	12.1	11.3	11.3
Sr ↔ O	30.5	28.8	31.8	29.6
Ta ↔ Zr	3.1	3.3	3.1	3.1
Ta ↔ O	40.3	41.0	30.6	40.3
Zr ↔ O	37.6	36.6	37.7	26.5

References

1. Chen, C. H. *et al.* Stable lithium-ion conducting perovskite lithium-strontium-tantalum- zirconium-oxide system. *Solid State Ionics* **167**, 263–272 (2004).
2. Kertesz, Z. I. *Lange's Handbook of Chemistry*. *Agronomy Journal* vol. 36 (Agronomy Journal, 1944).
3. Kimura, K., Wagatsuma, K., Tojo, T., Inada, R. & Sakurai, Y. Effect of composition on lithium-ion conductivity for perovskite-type lithium-strontium-tantalum-zirconium-oxide solid electrolytes. *Ceramics International* **42**, 5546–5552 (2016).
4. Luo, J., Zhong, S., Huang, Z., Huang, B. & Wang, C. High Li^{+}-conductive perovskite $Li_{3/8}Sr_{7/16}Ta_{3/4}Zr_{1/4}O_3$ electrolyte prepared by hot-pressing for all-solid-state Li-ion batteries. *Solid State Ionics* **338**, 1–4 (2019).
5. Inada, R., Kimura, K., Kusakabe, K., Tojo, T. & Sakurai, Y. Synthesis and lithium-ion conductivity for perovskite-type $Li_{3/8}Sr_{7/16}Ta_{3/4}Zr_{1/4}O_3$ solid electrolyte by powder-bed sintering. *Solid State Ionics* **261**, 95–99 (2014).
6. Polczyk, T., Zaja, W., Zia, M. & Konrad, S. Mitigation of grain boundary resistance in $La_{2/3-x}Li_{3x}TiO_3$ perovskite as an electrolyte for solid-state Li-ion batteries. *Journal of Materials Science* **56**, 2435–2450 (2021).
7. Mariappan, C. R., Yada, C., Rosciano, F. & Roling, B. Correlation between micro-structural properties and ionic conductivity of $Li_{1.5}Al_{0.5}Ge_{1.5}(PO_4)_3$ ceramics. *Journal of Power Sources* **196**, 6456–6464 (2011).
8. Xu, Q. *et al.* Insights into the reactive sintering and separated specific grain/grain boundary conductivities of $Li_{1.3}Al_{0.3}Ti_{1.7}(PO_4)_3$. *Journal of Power Sources* **492**, (2021).
9. Gönüllü, Y., Kelm, K., Mathur, S. & Saruhan, B. Equivalent circuit models for determination of the relation between the sensing behavior and properties of undoped/Cr doped TiO_2 NTs. *Chemosensors* **2**, 69–84 (2014).
10. Yu, L., Li, M., Wen, J., Amine, K. & Lu, J. (S)TEM-EELS as an advanced characterization technique for lithium-ion batteries. *Materials Chemistry Frontiers* **5**, 5186–5193 (2021).
11. Saitoh, M. *et al.* Systematic analysis of electron energy-loss near-edge structures in Li-ion battery materials. *Physical Chemistry Chemical Physics* **20**, 25052–25061 (2018).
12. Gao, X. *et al.* Cation ordering in A-site-deficient Li-ion conducting perovskites $La_{(1-x)/3}Li_xNbO_3$. *Journal of Materials Chemistry A* **3**, 3351–3359 (2015).
13. Inaguma, Y. & Nakashima, M. A rechargeable lithium-air battery using a lithium ion-conducting lanthanum lithium titanate ceramics as an electrolyte separator. *Journal of Power Sources* **228**, 250–255 (2013).
14. Zhao, Y. *et al.* A chemistry and material perspective on lithium redox flow batteries towards high-density electrical energy storage. *Chemical Society Reviews* **44**, 7968–7996 (2015).
15. Lian, P. J. *et al.* Inorganic sulfide solid electrolytes for all-solid-state lithium secondary batteries. *Journal of Materials Chemistry A* **7**, 20540–20557 (2019).
16. Huang, J. *et al.* Garnet-type solid-state electrolytes and interfaces in all-solid-state lithium batteries: progress and perspective. *Applied Materials Today* **20**, (2020).
17. Li, Y. *et al.* A Perovskite Electrolyte That Is Stable in Moist Air for Lithium-Ion Batteries. *Angewandte Chemie - International Edition* **57**, 8587–8591 (2018).
18. Christensen, J. *et al.* A Critical Review of Li/Air Batteries. *Journal of The Electrochemical Society* **159**, R1–R30 (2011).
19. Sun, Y. *et al.* Recent Progress in Lithium Lanthanum Titanate Electrolyte towards All Solid-State Lithium Ion Secondary Battery. *Critical Reviews in Solid State and Materials Sciences* **44**, 265–282 (2019).
